# Cortico-thalamic tremor circuits and their associations with deep brain stimulation effects in essential tremor

**DOI:** 10.1093/brain/awae387

**Published:** 2024-11-27

**Authors:** Shenghong He, Timothy O West, Fernando R Plazas, Laura Wehmeyer, Alek Pogosyan, Alceste Deli, Christoph Wiest, Damian M Herz, Thomas G Simpson, Pablo Andrade, Fahd Baig, Michael G Hart, Francesca Morgante, James J FitzGerald, Michael T Barbe, Veerle Visser-Vandewalle, Alexander L Green, Erlick A Pereira, Hayriye Cagnan, Huiling Tan

**Affiliations:** Medical Research Council Brain Network Dynamics Unit, University of Oxford, Oxford OX1 3TH, UK; Nuffield Department of Clinical Neurosciences, University of Oxford, Oxford OX3 9DU, UK; Medical Research Council Brain Network Dynamics Unit, University of Oxford, Oxford OX1 3TH, UK; Nuffield Department of Clinical Neurosciences, University of Oxford, Oxford OX3 9DU, UK; Department of Bioengineering, Imperial College London, London SW7 2AZ, UK; Medical Research Council Brain Network Dynamics Unit, University of Oxford, Oxford OX1 3TH, UK; Nuffield Department of Clinical Neurosciences, University of Oxford, Oxford OX3 9DU, UK; Medical Research Council Brain Network Dynamics Unit, University of Oxford, Oxford OX1 3TH, UK; Nuffield Department of Clinical Neurosciences, University of Oxford, Oxford OX3 9DU, UK; Department of Stereotactic and Functional Neurosurgery, University Hospital Cologne, Faculty of Medicine, University of Cologne, Cologne 50937, Germany; Medical Research Council Brain Network Dynamics Unit, University of Oxford, Oxford OX1 3TH, UK; Nuffield Department of Clinical Neurosciences, University of Oxford, Oxford OX3 9DU, UK; Medical Research Council Brain Network Dynamics Unit, University of Oxford, Oxford OX1 3TH, UK; Neuromodulation and Motor Control Section, Neurosciences and Cell Biology Institute, City St George’s, University of London, London SW17 0RE, UK; Medical Research Council Brain Network Dynamics Unit, University of Oxford, Oxford OX1 3TH, UK; Nuffield Department of Clinical Neurosciences, University of Oxford, Oxford OX3 9DU, UK; Medical Research Council Brain Network Dynamics Unit, University of Oxford, Oxford OX1 3TH, UK; Nuffield Department of Clinical Neurosciences, University of Oxford, Oxford OX3 9DU, UK; Movement Disorders and Neurostimulation Section, Department of Neurology, Focus Program Translational Neuroscience (FTN), University Medical Center of the Johannes Gutenberg-University Mainz, Mainz 55131, Germany; Medical Research Council Brain Network Dynamics Unit, University of Oxford, Oxford OX1 3TH, UK; Nuffield Department of Clinical Neurosciences, University of Oxford, Oxford OX3 9DU, UK; Department of Stereotactic and Functional Neurosurgery, University Hospital Cologne, Faculty of Medicine, University of Cologne, Cologne 50937, Germany; Neuromodulation and Motor Control Section, Neurosciences and Cell Biology Institute, City St George’s, University of London, London SW17 0RE, UK; Neuromodulation and Motor Control Section, Neurosciences and Cell Biology Institute, City St George’s, University of London, London SW17 0RE, UK; Neuromodulation and Motor Control Section, Neurosciences and Cell Biology Institute, City St George’s, University of London, London SW17 0RE, UK; Nuffield Department of Clinical Neurosciences, University of Oxford, Oxford OX3 9DU, UK; Nuffield Department of Surgical Sciences, University of Oxford, Oxford OX3 9DU, UK; Department of Neurology, University Hospital Cologne, Faculty of Medicine, University of Cologne, Cologne 50937, Germany; Department of Stereotactic and Functional Neurosurgery, University Hospital Cologne, Faculty of Medicine, University of Cologne, Cologne 50937, Germany; Nuffield Department of Clinical Neurosciences, University of Oxford, Oxford OX3 9DU, UK; Nuffield Department of Surgical Sciences, University of Oxford, Oxford OX3 9DU, UK; Neuromodulation and Motor Control Section, Neurosciences and Cell Biology Institute, City St George’s, University of London, London SW17 0RE, UK; Medical Research Council Brain Network Dynamics Unit, University of Oxford, Oxford OX1 3TH, UK; Nuffield Department of Clinical Neurosciences, University of Oxford, Oxford OX3 9DU, UK; Department of Bioengineering, Imperial College London, London SW7 2AZ, UK; Medical Research Council Brain Network Dynamics Unit, University of Oxford, Oxford OX1 3TH, UK; Nuffield Department of Clinical Neurosciences, University of Oxford, Oxford OX3 9DU, UK

**Keywords:** essential tremor, deep brain stimulation, efferent and afferent, directed connectivity, local field potential

## Abstract

Essential tremor is one of the most common movement disorders in adults. Deep brain stimulation (DBS) of the ventralis intermediate nucleus of the thalamus and/or the posterior subthalamic area has been shown to provide significant tremor suppression in patients with essential tremor, but with significant inter-patient variability and habituation to the stimulation. Several non-invasive neuromodulation techniques targeting other parts of the CNS, including cerebellar, motor cortex or peripheral nerves, have also been developed for treating essential tremor, but the clinical outcomes remain inconsistent. Existing studies suggest that pathology in essential tremor might emerge from multiple cortical and subcortical areas, but its exact mechanisms remain unclear.

By simultaneously capturing neural activities from motor cortices and thalami and recording hand tremor signals via accelerometers in 15 human subjects who had undergone lead implantations for DBS, we systematically characterized the efferent and afferent cortico-thalamic tremor networks. Through the comparisons of these network characteristics and tremor amplitude between DBS off and on conditions, we also investigated the associations between different tremor network characteristics and the magnitude of the DBS effect.

Our findings implicate the thalamus, specifically the contralateral hemisphere, as the primary generator of tremor in essential tremor, also with a significant contribution of the ipsilateral hemisphere. Although there is no direct correlation between the cortico-tremor connectivity and tremor power or reduced tremor by DBS, the strength of connectivity from the motor cortex to the thalamus and vice versa at tremor frequency predicts baseline tremor power and effect of DBS. Interestingly, there is no correlation between these two connectivity pathways themselves, suggesting that, independent of the subcortical pathway, the motor cortex appears to play a relatively distinct role, possibly mediated through an afferent/feedback loop in the propagation of tremor. DBS has a greater clinical effect in those with stronger cortico-thalamo-tremor connectivity involving the contralateral thalamus, which is also associated with bigger and more stable tremor measured with an accelerometer. Interestingly, stronger cross-hemisphere coupling between left and right thalami is associated with more unstable tremor.

This study provides important insights for a better understanding of the cortico-thalamic tremor-generating network and its implication for the development of patient-specific therapeutic approaches for essential tremor.

## Teaser

He *et al.* recorded neural activity from the motor cortex and thalamus, along with hand tremor signals, in people with essential tremor. Their findings provide new insights into the cortico-thalamic network involved in tremor generation, and have important implications for the development of patient-specific therapeutic approaches.

## Introduction

Essential tremor (ET) is one of the most common movement disorders in adults, with an estimated prevalence of 0.5%–5%.^1-3^ Based on a series of cortico-cortical, cortico-muscular, and intermuscular coherence analyses, Raethjen *et al*.^4-6^ proposed that tremor in ET emerges from a number of cortical and subcortical motor centres, with each node acting as a dynamically changing oscillator and temporarily entraining each other. In line with this theory, various neuromodulation techniques targeting distinct brain regions or other components of the CNS have been used clinically or experimentally to treat ET. In clinical practice, high-frequency continuous deep brain stimulation (DBS) specifically targeting the ventralis intermediate nucleus (VIM) of the thalamus has been widely used and demonstrated significant efficacy in suppressing tremor in patients with ET. Additionally, alternative targets, such as the posterior subthalamic area [PSA, including zona incerta (ZI)], have also been proposed.^7-11^ However, despite these promising clinical outcomes, notable inter-patient variability and habituation to the stimulation have been observed. In the realm of experimental non-invasive neuromodulation, several techniques have been developed for treating ET. These include transcranial alternating/direct current stimulation targeting cerebellar^12-14^ or motor cortex,^15^ repetitive transcranial magnetic stimulation targeting cerebellar^16-18^ or motor cortex,^19,20^ and electrical stimulation targeting peripheral nerves,^21,22^ although the clinical outcomes remain inconsistent. To optimize the efficacy of both invasive and non-invasive neuromodulatory approaches, a more precise understanding of the underlying mechanisms driving tremor in ET is needed. This entails elucidating the intricate interplay of multiple cortical and subcortical brain regions involved in the pathophysiology of ET.^4-6^ However, most of the existing studies are based on recordings from only a single node in the motor circuit (cortical or subcortical) and lack within-subject pre- and post-intervention comparisons. Thus, the characteristics of cortical- and subcortico-tremor networks and how they change with intervention targeting the relevant nodes are still unclear.

In this study, based on the simultaneous recording of cortical EEG, thalamic local field potentials (LFPs) and limb acceleration measurements from patients with ET, we characterized cortico-thalamo-tremor networks through a directed connectivity analysis called generalized orthogonalized partial directed coherence (gOPDC)^23^ and explored the associations between cortico-thalamo-tremor network characteristics and hand tremor characteristics. Furthermore, based on the data recorded during DBS off and DBS on from each individual participant, we also investigated how the cortico-thalamo-tremor network characteristics predict DBS effect in tremor suppression.

## Materials and methods

### Human subjects and experimental protocol

**Figure 1:**
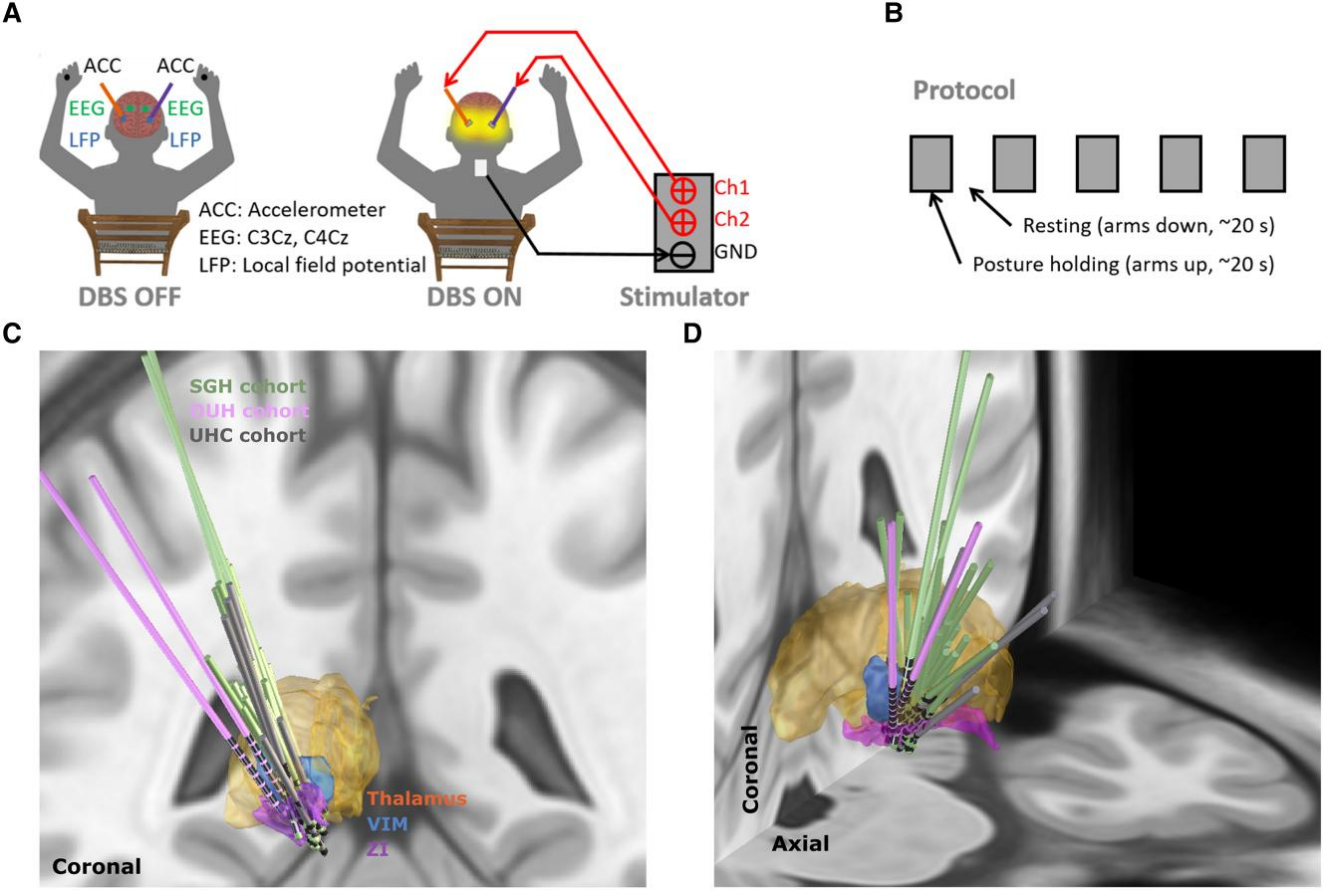
**Experimental protocol.** (**A**) Schematic diagram of the posture-holding task performed when the DBS is switched off (*left*) and on (*right*). (**B**) Time line for the experimental protocol, which consists of 10 posture-holding blocks (∼20 s per block) when both arms are raised up and 10 resting blocks when both arms are put down. (**C** and **D**) 3D reconstruction in coronal (**C**) and coronal–axial (**D**) views of all analysed DBS leads localized in standard Montreal Neurological Institute (MNI)-152_2009b space using Lead-DBS.^25,26^ Electrodes in the left hemisphere were mirrored to the right hemisphere. ACC = accelerometer; Ch = channel; DBS = deep brain stimulation; GND = ground; LFP = local field potential; OUH = Oxford University Hospital; SGH = St George’s Hospital; UHC = University Hospital Cologne; VIM = ventral intermediate thalamus; ZI = zona incerta.

Fifteen patients (mean age = 69.1 ± 7.26 years; mean disease duration = 21.1 ± 14.5 years; six females) with ET who underwent DBS surgery participated in this study [Patients 1–7 and 12 were published previously].^24^ All participants underwent bilateral implantations of DBS electrodes targeting the VIM thalamus and/or PSA/ZI area. The experimental protocol involved a posture-holding task performed while sitting comfortably in a chair, with both arms raised up to the height of shoulders (Fig. 1A). The task was performed in blocks in both DBS off and on conditions, with each block lasting ∼20 s. There was a resting period when both arms were put down between two posture-holding blocks (Fig. 1B). On average, the posture-holding task was performed for 195.9 ± 11.5 s [mean ± standard error of the mean (SEM)] and 196.7 ± 14.8 s in DBS off and on conditions, respectively. The study was approved by the local ethics committees, and all participants provided their informed written consent according to the Declaration of Helsinki. Clinical details of all participants are summarized in Table 1.

**Table 1:** Clinical details of all recorded participants

Patient	Sex	Age (years)	DD (years)	DBS lead	L/R amplitude (mA)	Centre	DBS target	Diagnosis	Predominant symptom(s) before surgery	Preoperative medication
1^a,b^	F	77	21	Abb	1.1/NA	SGH	VIM-PSA	ET	Tremor, gait ataxia, tremor worse on right, upper limb and voice tremor	Half Sinemet CR 125 mg at night
2^a,b^	M	61	20	Abb	NA/3	SGH	VIM-PSA	ET	Tremor, dystonia, upper limb tremor and head tremor	None for tremor, previously primidone, propranolol, gabapentin, levodopa
3	M	75	18	Abb	2.5/2.0	SGH	VIM-PSA	ET	Tremor, upper limb, lower limb and head tremor	None for tremor, previously tried primidone, clonazepam, propranolol, gabapentin, topiramate
4	M	70	8	Abb	1.8/1.8	SGH	VIM-PSA	ET	Tremor, upper limb, with right worse than left, lower limb tremor	None for tremor, previously tried propranolol, gabapentin, topiramate, lamotrigine, primidone
5	F	62	45	Abb	2/2	SGH	VIM-PSA	ET	Tremor, upper limb tremor left worse than right, voice tremor	None for tremor, previously propranolol, pregabilin, primidone
6	M	70	5	Abb	3/3	SGH	VIM-PSA	ET	Tremor, upper limb left worse than right	None for tremor, previously pregabalin, primidone, propranolol, topiramate, gabapentin
7	M	67	47	Abb	1.5/1.5	SGH	VIM-PSA	ET	Tremor, upper limb right worse than left, head tremor	None for tremor, previously tried popranolol, topiramate, gabapentin
8^b^	M	76	50	Abb	2.0/2.0	SGH	VIM-PSA	ET	Upper limb action tremor (left > right)	Propranolol, primidone, diazepam
9^b^	F	77	14	Abb	2.0/2.0	SGH	VIM-PSA	ET	Upper and lower limb tremor (right > left)	Propranolol, primidone, diazepam
10	F	79	20	Bos^1^	2.0/1.5	SGH	VIM-PSA	ET	Upper limbs tremor (right > left)	Propranolol, topiramate, primidone
11	M	73	15	Abb	1.0/1.0	SGH	VIM-PSA	ET	Upper limbs tremor (right > left)	Propranolol, primidone
12	F	65	UN	Bos^2^	1.1/1.5	OUH	VIM	ET	Tremor, upper limb, worse intention tremor on left	None for tremor
13^b^	F	58	15	Med	1.5/1.5	UHC	VIM	ET	Tremor in both hands (left > right)	None pre-operatively, previous primidone therapy was unsuccessful
14^a,b^	M	55	8	Bos^3^	NA/2.0	UHC	VIM	ET	Tremor left hand	Previously propranolol, primidone, levetiracetam and gabapentin
15	M	72	10	Med	3.5/1.2	UHC	VIM	ET	Tremor in both hands (right > left), head tremor	Previously propranolol, mylepsinum and gabapentin
Mean	–	69.1	21.1	–	1.85	–	–	–	–	–
SD	–	7.26	14.5	–	0.56	–	–	–	–	–

Abb = Abbott infinity 1.5 mm spaced directional leads (1–4), Abbott; Amp = amplitude; Bos^1^ = Boston Cartesia™ HX leads with 3-3-3-3-1-1-1-1 configuration, Boston Scientific; Bos^2^ = Boston linear 8 contact leads (1–8), Boston Scientific; Bos^3^ = Boston Vercise™ directional lead with 1-3-3-1 configuration, Boston Scientific; DBS = deep brain stimulation; DD = disease duration; ET = essential tremor; F = female; L = left; M = male; Med = Medtronic SenSight™ directional leads; NA = not applicable; OUH = Oxford University Hospital; R = right; PSA = posterior subthalamic area; SGH = St George’s Hospital; UHC = University Hospital Cologne; VIM = ventral intermediate thalamus; SD = standard deviation.

^a^Only unilateral DBS was applied.

^b^Tremor from only one hand was recorded; Patient 1 had gait ataxia, which is sometimes seen in advanced ET. Patient 2 had an overlap between ET and dystonic tremor.

### Stimulation

Stimulation was applied bilaterally (except for Patients 1, 2 and 14, who received unilateral stimulation contralateral to the tremor-dominant hand) using a highly configurable custom-built neurostimulator or a European Conformity (CE) marked stimulator. In this study, monopolar stimulation was delivered with a fixed stimulation frequency of 130 Hz, a pulse width of 60 µs and an interphase gap of 20 µs. These parameters are illustrated in Supplementary Fig. 1. The stimulation reference was connected to an electrode patch attached to the back of the participant (Fig. 1A). These stimulation parameters and configurations were selected based on previous literature.^24,27-33^ The stimulation contact was selected as follows. First, contact levels targeting the VIM-PSA area based on imaging data and/or feedback from neurosurgeon after operation were considered. Second, among them, a contact-searching procedure was applied to select the final stimulation contact for each hemisphere. Specifically, we delivered continuous DBS initially at 0.5 mA, then increased the amplitude progressively in 0.5 mA increments until clinical benefit was seen without side effects, such as paraesthesia, or until 3.5 mA was reached as the maximum amplitude. On average, the amplitude used in this study was 1.89 ± 0.12 mA (mean ± SEM). Details of the stimulation configuration for each participant are summarized in Table 1.

### Data recording

Recordings from 15 participants were conducted 1–5 days after the electrode implantation, when the DBS leads were temporarily externalized. While performing the posture-holding task illustrated in Fig. 1, bilateral LFPs, EEGs covering ‘Cz’, ‘C3’, ‘C4’, ‘CPz’, ‘CP3’ and ‘CP4’ according to the standard 10–20 system, and limb accelerations acquired using tri-axial accelerometers taped to the back of both hands were recorded simultaneously using a Porti (TMS International) amplifier at a sampling rate of 2048 Hz (for P1–P7 and P12) or a Saga amplifier (TMS International) at a sampling rate of 4096 Hz (for P8–P11 and P13–P15). When a Porti amplifier was used, the segmented contacts were first constructed in ring mode, then LFPs from two adjacent levels or two levels neighbouring the stimulation contact were recorded in the differential bipolar mode to avoid saturation during stimulation. In contrast, LFPs from each individual contact were recorded in monopolar mode when a Saga amplifier was used, because it has a much higher tolerance of DC offset that might induce saturation during stimulation. Owing to lack of tremor on the other hand after DBS surgery, limb accelerations were recorded from only one hand for 6 (Patients 1, 2, 8, 9, 13 and 14) of the 15 participants (Table 1), resulting in 24 tremulous upper limbs.

### Data analysis

#### Preprocessing

**Figure 2:**
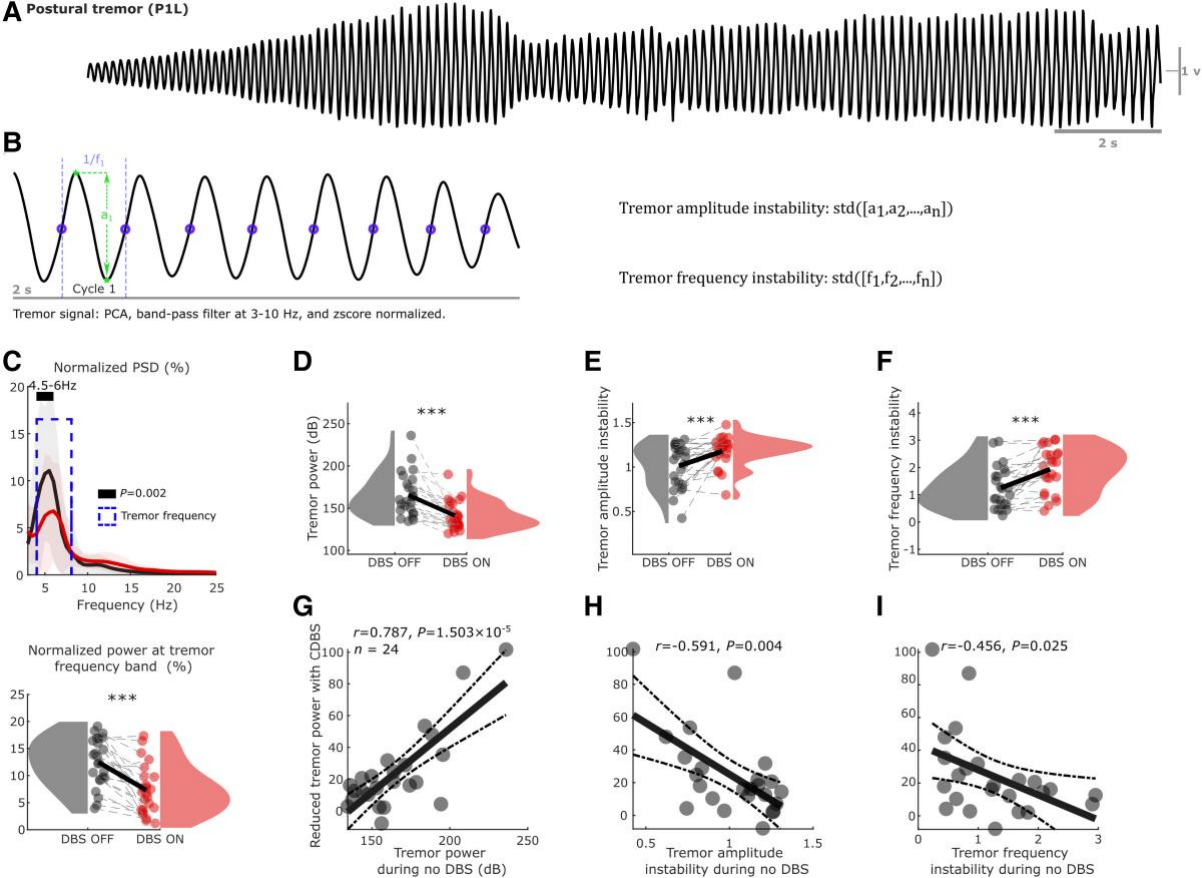
**Comparisons of tremor characteristics between DBS off and DBS on conditions.** (**A**) An example of 30 s postural tremor (P1L), showing the instability of tremor in essential tremor. (**B**) Demonstration of the quantifications of tremor amplitude and frequency instability from a segment of 2 s measurement from an accelerometer. (**C**) Normalized PSD of accelerometer measurements showed peaks at tremor frequency band in both DBS off (black) and DBS on (red) conditions (*top*), with a significant reduction of the normalized power (as a percentage) in the individualized tremor frequency band during DBS on (*bottom*). (**D**–**F**) Comparisons of tremor power (**D**), amplitude instability (**E**) and frequency instability (**F**) between DBS off (black) and DBS on (red) conditions using raincloud plots.^35^ Here, the shaded areas indicate distributions (probability density) of the data. (**G**–**I**) Tremor power during DBS off (baseline) is positively correlated (**G**) whereas tremor amplitude (**H**) and frequency (**I**) instability are negatively correlated with the reduction in tremor power during DBS (Pearson correlation). Solid lines in **C** and bars in **C**–**F** indicate the mean, while shaded areas in **C** and error bars in **C**–**F** indicate the standard error of the mean. Statistics were applied between DBS off and DBS on conditions using a non-parametric cluster-based permutation procedure in **C** (PSD) on a hand-by-hand basis, or using generalized linear mixed-effect modelling in all bar plots (**C**–**F**) on a trial-by-trial basis. Multiple comparisons were corrected by controlling the false discovery rate. ***P < 0.001 after false discovery rate correction. CDBS = continuous deep brain stimulation; DBS = deep brain stimulation; P1L = participant1, left hand; PCA = principal component analysis; PSD = power spectral density; std = standard deviation.

For the LFPs recorded in monopolar mode, bipolar signals were achieved offline by differentiating the recordings from two adjacent contacts or two contacts neighbouring the stimulation contact. In the cases with directional leads, only the contact pairs facing the same direction were considered. For the recorded EEGs, bipolar signals were constructed offline by differentiating between ‘C3’ and ‘Cz’ (i.e. ‘C3Cz’) or ‘C4’ and ‘Cz’ (i.e. ‘C4Cz’). The bipolar LFPs, EEGs and the recorded acceleration measurements were band-pass filtered at 1–95 Hz, then band-stop filtered at 48–52 Hz using two fourth order zero-phase Butterworth IIR digital filters in MATLAB (R2023-b, MathWorks). After filtering, a principal component analysis (PCA) was applied on the tri-axial acceleration measurements, and the first component was selected as the measurement of tremor on a given hand. PCA components reflect a linear combination of the three (orthogonal) axes, with the first component reflecting the orientation that captures the maximum variance in the data. This technique has precedence in previous studies.^13,34^ To consider the natural intra-individual tremor variability during posture holding (Fig. 2A), we split the data into non-overlapping 2 s segments and considered each segment as a trial. This procedure resulted in 98.0 ± 5.8 (mean ± SEM) and 98.3 ± 7.4 trials per subject in DBS off and DBS on conditions, respectively.

#### Spectral analysis

After preprocessing, power spectral density (PSD) was estimated using Welch’s overlapped segment averaging estimator for LFPs, EEGs and acceleration measurements of each individual in each trial,^36^ in a frequency range of 1–95 Hz with a 0.5 Hz resolution. To select the tremor frequency for each hand in each trial, we first normalized the PSD of the acceleration measurement against the sum of the power between 1 and 25 Hz, then the frequency between 3 and 10 Hz that has the maximum power was selected as the tremor frequency. To select one bipolar LFP for each hemisphere, we averaged the normalized PSD across trials for each bipolar LFP channel and selected the one with maximum power at the averaged tremor frequency of both tremor hands. Furthermore, for each trial (i.e. 2 s segment), the normalized PSD and power (raw and normalized) at the tremor frequency were calculated for EEGs, acceleration measurements and the selected bipolar LFPs for further analysis.

#### Tremor instability analysis

After preprocessing, tremor amplitude and frequency instability in each trial were quantified for each hand. Specifically, the acceleration measurements were high- and low-pass filtered at 3 and 10 Hz using two sixth-order zero-phase Butterworth IIR digital filters and *z*-score normalized. Then, zero-crossing points from negative to positive were used to identify the individual tremor cycle within each trial. For each tremor cycle, the instantaneous tremor amplitude was quantified as the distance between the peak and trough, while instantaneous tremor frequency was defined as the reciprocal of the duration of the tremor cycle, as shown in Fig. 2B. Finally, tremor amplitude and frequency instability were quantified as the standard deviation of the instantaneous tremor amplitude and frequency across cycles. Please note that with *z*-score normalization, these represent how stable the tremor is in terms of amplitude and frequency within the 2 s segment, as demonstrated in Supplementary Fig. 2. The tremor stability index^13,34^ and multiscale entropy^37^ have previously been proposed to distinguish ET and Parkinsonian tremor. Thus, these measurements were also computed for comparison.

#### Connectivity analysis

Based on the simultaneously recorded cortical, subcortical and tremor signals, we investigated the cortico-thalamo-tremor network characteristics through a directional connectivity analysis using a method called gOPDC.^23,38^ In this method, signal power was first orthogonalized before quantifying coherence to mitigate the effect of volume conduction.^39^ Briefly, a coefficient of a multivariate autoregressive model was converted to the spectral domain using the Fourier transform, then used to calculate the PSD matrix. Prior to frequency domain conversion, the multivariate autoregressive coefficients were orthogonalized.^38^ This effectively minimizes shared variance between the autoregressive components of the signals, such that correlations arise from off-diagonal terms (i.e. connectivity). Only the imaginary part of the orthogonalized partial directed coherence (OPDC) was considered to reduce spurious correlations introduced by factors such as movement/tremor artefact. In addition, the scale-invariant version of the classical partial directed coherence (i.e. gOPDC) was used to handle numerical problems associated with different variance of signal amplitudes in LFPs, EEGs and acceleration measurements (known as time-series scaling).^40,41^ This method has been shown to detect event-related directional information flow reliably at ∼10 Hz based on non-overlapping 1 s segments of neonatal EEGs.^23^ In the present study, we are mainly interested in the tremor frequency band at 3–8 Hz, hence the data were truncated into 2 s non-overlapping segments. Based on gOPDC, the mean efferent (from cortices/thalamus to tremor) and afferent (from tremor back to cortices/thalamus) connectivity in a frequency range covering 2 Hz around the basic tremor frequency and 2 Hz around the second harmonic frequency were analysed. Furthermore, direct and indirect causal effects of a certain structure were explored by comparing the unconditioned versus conditioned gOPDC models, i.e. excluding or including the corresponding source.^23^ Each gOPDC measurement was compared against its surrogate distribution. To this end, the preprocessed continuous tremor time series was divided into two segments according to a randomly selected point (with a minimum of 2 s margin on each side), then swapped back and forth to disrupt the coupling between EEG/LFP and tremor signals. Then, the shuffled data were truncated into non-overlapping 2 s trials. This procedure was repeated until we obtained 1000 trials of shuffled data. The same gOPDC metrics were derived from the shuffled data, resulting in a surrogate distribution of 1000 values per measurement.^42^ This approach ensured that any signatures of connectivity remaining, following disruption of the EEG/LFP and tremor signal pairs, arose from the independent statistics of each signal.

#### Spatial distributions of the connectivity measurements

Lead placements were confirmed by fusion of preoperative MRI and postoperative CT scans, which were established further by reconstructing the electrode trajectories and location of different contacts using the Lead-DBS MATLAB toolbox (version 2.6.0).^25^ The electrode locations were registered and normalized into the Montreal Neurologic Institute (MNI) 152–2009b space using the Connectomic ET Target Atlas.^11^ As shown in Fig. 1C and D, most of the tested electrodes targeted the VIM-PSA area. To investigate the spatial distributions of the bidirectional gOPDC connectivity (thalamo-cortical and cortico-thalamic) and their associations with different targets for ET, we repeated the connectivity analyses for all available bipolar LFP channels from all patients and mapped them onto the MNI space based on the coordinates of each contact. In addition, for each hemisphere, the volume of tissue activated (VTA) during stimulation was estimated using a finite element method,^25^ based on the individual electrode position used for the connectivity calculation and a common stimulation amplitude (i.e. 1 mA). Subsequently, the intersections between the VTA and different subcortical structures (e.g. VIM and ZI) were quantified and used to correlate with different connectivity measurements.

### Statistical analysis

Statistical analyses were conducted using custom-written scripts in MATLAB R2023-b (The MathWorks Inc., Nantucket, MA, USA).

To compare the PSD of EEGs, LFPs and acceleration measurements between DBS off and DBS on conditions, a non-parametric cluster-based permutation procedure (repeated 2000 times) was applied, in which multiple comparisons were controlled theoretically.^43^

To compare the tremor characteristics (power, amplitude instability and frequency instability) or gOPDC measurements quantified on a trial-by-trial basis between different conditions (e.g. DBS off versus DBS on, unconditioned versus conditioned gOPDC models, or real gOPDC versus its null distribution), generalized linear mixed-effect (GLME) modelling was used.^44,45^ We also used GLME to investigate the associations between gOPDC measurements and tremor characteristics further on a trial-by-trial basis. In each GLME model, the slope(s) between the predictor(s) and the dependent variable were set to be fixed across all tremor hands, and a random intercept was set to vary by hand. The parameters were estimated based on maximum-likelihood using Laplace approximation. The Akaike information criterion, estimated value with standard error of the coefficient (*k* ± SE), multiple comparisons corrected *P*-value, and proportion of variability in the response explained by the fitted model (*R*^2^) were reported. Here, multiple comparisons applied to different measurements were corrected using false discovery rate approach.^46,47^

To explore the correlations between different tremor characteristics or gOPDC measurements and the effect of DBS in tremor suppression, or between different gOPDC measurements, Pearson correlation was applied on a hand-by-hand basis. For each correlation analysis, the pairwise linear correlation coefficient (*r*), multiple comparisons corrected *P*-value (based on false discovery rate) and sample size (*n*) were reported. Here, the sample size was equal to the number of tremulous upper limbs (*n* = 24), unless outliers were identified according to the Pauta criterion (3σ criterion).

## Results

### Continuous DBS reduces tremor power and stability, and the DBS effect is correlated with baseline tremor power and instability

The amplitude of postural tremor in ET is unstable over time,^13,48-50^ as shown in Fig. 2A, which motivated us to quantify tremor characteristics, including power at tremor frequencies (peak frequency ± 1 Hz), tremor amplitude instability, and frequency instability in non-overlapping 2 s epochs, as shown in Fig. 2B. As expected, there was a significant reduction in tremor power during DBS on compared with DBS off (Fig. 2C, PSD at 4.5–6 Hz: *t* = 3.799, *P* = 0.002; normalized tremor power: *k* = −5.280 ± 0.120, *P* < 1 × 10^−4^; Fig. 2D, absolute tremor power: *k* = −26.502 ± 0.621, *P* < 1 × 10^−4^), although tremor-frequency peaks were identified in both DBS off and DBS on conditions. This was accompanied by a significant reduction in power at the tremor frequency band in the VIM thalamic LFPs (Supplementary Fig. 3A and B) and cortical EEGs (Supplementary Fig. 3C and D). In addition, DBS significantly increased the instabilities of tremor amplitude (Fig. 2E, *k* = 0.173 ± 0.011, *P* < 1 × 10^−4^) and frequency (Fig. 2F, *k* = 0.744 ± 0.029, *P* < 1 × 10^−4^). Here, *k* indicates estimated value with standard error of the coefficient using GLME modelling. Apart from an expected positive correlation between the level of tremor reduction with DBS and the baseline tremor power during DBS off (Fig. 2G, *r* = 0.787, *P* = 1.50 × 10^−5^), baseline tremor instability was also found to be negatively correlated with the effect of DBS (Fig. 2H, amplitude instability, *r* = −0.591, *P* = 0.004; Fig. 2I, frequency instability, *r* = −0.456, *P* = 0.025). We repeated this analysis using two other tremor instability measurements, namely tremor stability index^13,34^ and multiscale entropy.^37^ As shown in Supplementary Fig. 4, these measurements were highly correlated with each other and showed similar relationships with respect to the effect of DBS. Together, these findings suggested that more severe and stable tremor during DBS off was associated with a larger effect of DBS on tremor reduction.

### The efferent and afferent thalamic-tremor networks are both lateralized and interact across hemispheres

**Figure 3:**
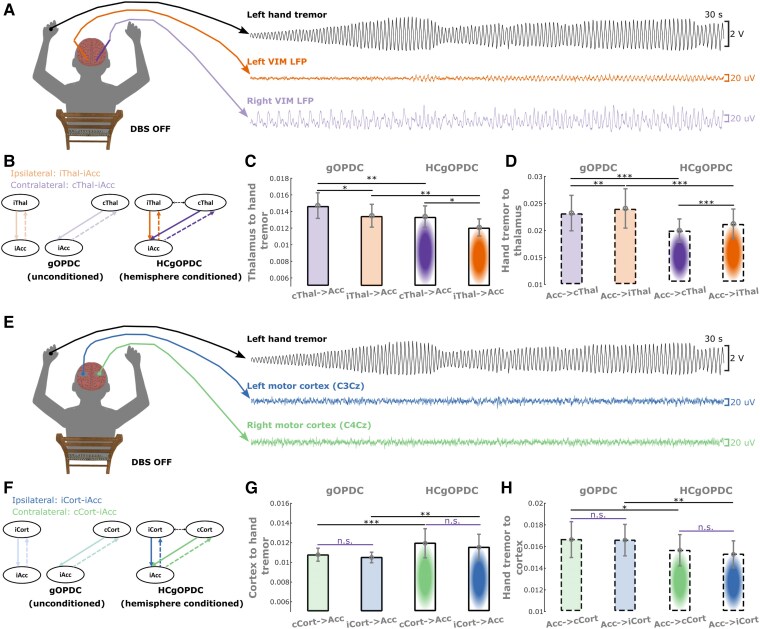
**Characteristics of thalamic-tremor and cortico-tremor networks when DBS was switched off.** (**A**) A demonstration of left-hand postural tremor and thalamic LFP recordings from Participant 1, left hand (P1L) during DBS off condition. (**B**) Directed connectivity between VIM thalamus and hand tremor quantified using gOPDC. Solid lines indicate efferent connectivity from thalamus to hand tremor, while dashed lines indicate afferent connectivity from hand tremor to thalamus. Orange and purple represent the connectivity with ipsilateral and contralateral VIM thalami, respectively. The *top* and *bottom* panels indicate gOPDC involving only one thalamus (unconditioned) and both thalami (HC), respectively. (**C**) Efferent connectivity from the contralateral thalamus was significantly stronger than that from the ipsilateral hemisphere in both unconditioned (*left*) and HC (*right*) models. When conditioning the impact from the other hemisphere, the efferent connectivities from the contralateral (purple) and ipsilateral (orange) thalami to hand tremor were both significantly reduced. (**D**) Afferent connectivity from hand tremor to the contralateral thalamus was significantly weaker than that to the ipsilateral hemisphere in both unconditioned (*left*) and hemisphere-conditioned (*right*) models. When conditioning the impact from the other hemisphere, the afferent connectivities from hand tremor to the contralateral (purple) and ipsilateral (orange) thalami were both significantly reduced. (**E**–**H**) The same as **A**–**D** but for cortico-tremor network. Bars and error bars indicate mean and standard error of the mean, respectively. Statistics were applied on each comparison using generalized linear mixed-effect modelling on a trial-by-trial basis. Multiple comparisons were corrected by controlling the false discovery rate. *P < 0.05, **P < 0.01 and ***P < 0.001, after false discovery rate correction. Acc = accelerometer; cCort = contralateral motor cortex; cThal = contralateral thalamus; DBS = deep brain stimulation; gOPDC = generalized orthogonalized partial directed coherence; HCgOPDC = hemisphere-conditioned generalized orthogonalized partial directed coherence; iCort = ipsilateral motor cortex; iThal = ipsilateral thalamus; LFP = local field potential; VIM = ventral intermediate thalamus.

Based on the simultaneously recorded hand-acceleration measurements and bilateral thalamic LFPs during posture holding (Fig. 3A), we characterized bidirectional connectivity between VIM thalamus and hand tremor in the tremor frequency band using gOPDC. As shown in Supplementary Table 1, we first tested the main effects of laterality (contralateral versus ipsilateral), cross-hemisphere coupling (conditioned versus unconditioned) and directionality (efferent versus afferent), in addition to the interaction effects between them. This analysis revealed significant main effects for all these conditions and significant interaction effects between laterality and directionality, and between cross-hemisphere coupling and directionality. We then conducted pairwise comparisons, and the results revealed that without DBS, the efferent connectivity from the contralateral thalamus to hand tremor was significantly stronger than that from the ipsilateral thalamus (Fig. 3C, unconditioned model, *k* = −0.001 ± 0.001, *P* = 0.029; hemisphere-conditioned model, *k* = −0.001 ± 0.001, *P* = 0.011), as expected. However, the afferent network showed an opposite pattern, with a significantly stronger input from hand tremor to the ipsilateral thalamus than that to the contralateral thalamus (Fig. 3D, unconditioned model, *k* = 0.002 ± 0.001, *P* = 0.001; hemisphere-conditioned model, *k* = 0.003 ± 0.001, *P* = 4.73 × 10^−5^). Overall, the strength of the afferent network was stronger than the efferent network. This thalamic-tremor network laterality disappeared during DBS (Supplementary Fig. 5). Compared with the model involving only unilateral (either contralateral or ipsilateral) thalamus and hand tremor (Fig. 3B, left, unconditioned model), conditioning the impact from the other thalamus (hemisphere-conditioned model, Fig. 3B*, right*) significantly reduced the efferent connectivity from both the contralateral (Fig. 3C, *k* = −0.002 ± 0.001, *P* = 0.004) and ipsilateral (Fig. 3C, *k* = −0.002 ± 0.001, *P* = 0.002) thalami to hand tremor. Likewise, the afferent connectivity from hand tremor to both the contralateral (Fig. 3D, *k* = −0.004 ± 0.001, *P* = 7.88 × 10^−11^) and ipsilateral (Fig. 3D, *k* = −0.004 ± 0.001, *P* = 2.91 × 10^−8^) thalami was also significantly reduced in the hemisphere-conditioned model compared with the unconditioned model. This suggests that there was cross-hemisphere coupling between the two thalami in the thalamic-tremor network. During DBS, the hemisphere-conditioned model also significantly reduced the efferent connectivity from both thalami to hand tremor, but not the afferent connectivity from hand tremor to both thalami (Supplementary Fig. 5). The details of the GLME models used for these tests are summarized in Supplementary Table 1.

### The efferent and afferent cortico-tremor networks are non-lateralized but interact across hemispheres

Likewise, we characterized bidirectional (efferent and afferent) connectivity between cortical activities and hand tremor in the tremor frequency band using gOPDC (Fig. 3E). We first identified significant main effects on cross-hemisphere coupling and directionality, but not on laterality. The interaction between cross-hemisphere coupling and directionality was also significant (Supplementary Table 2). We then conducted pairwise comparisons, and the results revealed that without DBS there was no significant difference between the efferent connectivity from the contralateral and ipsilateral motor cortices to hand tremor in either the unconditioned model (Fig. 3G) or the hemisphere-conditioned model. Similar results were observed in the afferent tremor to cortical connectivity (Fig. 3H). Compared with the model involving only unilateral sensorimotor cortex and hand tremor (Fig. 3F, left, unconditioned model), conditioning the impact from the other cortex (conditioned model, Fig. 3F, right) significantly increased the efferent connectivity from both the contralateral (Fig. 3G, *k* = 0.001 ± 4 × 10^−4^, *P* = 9.0 × 10^−4^) and ipsilateral (Fig. 3G, *k* = 0.001 ± 4 × 10^−4^, *P* = 0.003) sensorimotor cortices to hand tremor. However, the afferent connectivity from hand tremor to both the contralateral (Fig. 3H, *k* = −0.001 ± 0.001, *P* = 0.030) and ipsilateral (Fig. 3H, *k* = −0.001 ± 4 × 10^−4^, *P* = 0.007) cortices reduced significantly in the conditioned model compared with the unconditioned model. During DBS, none of these comparisons was significant (Supplementary Fig. 6). These results suggest that the cortico-tremor network is not lateralized but interacts across hemispheres; in other words, there is coupling between the ipsilateral and contralateral cortices, and both of them contribute to hand tremor equally. The details of the GLME models used for these tests are summarized in Supplementary Table 2.

### Interaction between the thalamic-tremor and cortico-tremor networks

**Figure 4:**
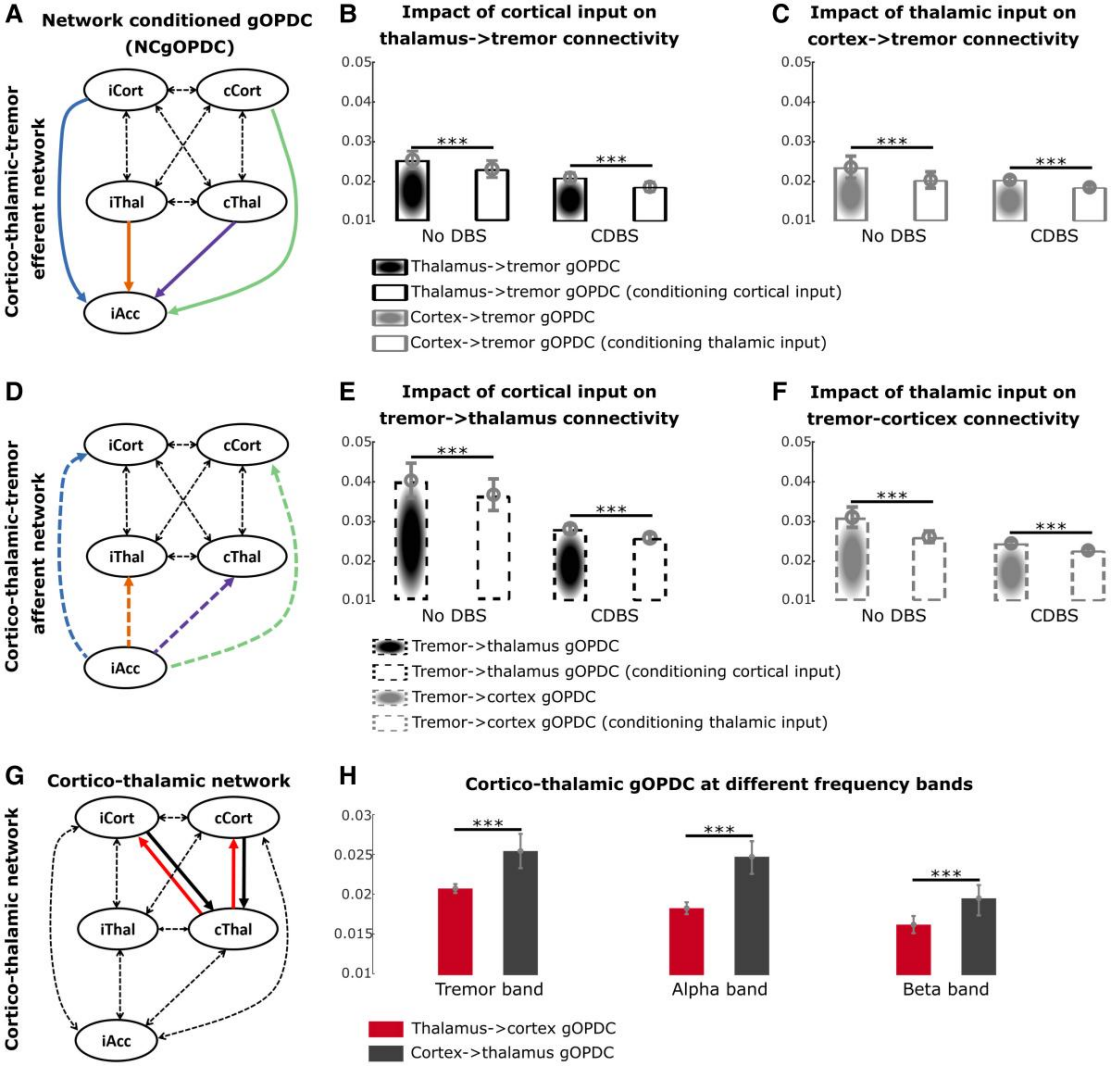
**Characteristics of cortico-thalamo-tremor network.** (**A**) Directed efferent connectivity from sensorimotor cortex and VIM thalamus to hand tremor quantified using gOPDC. (**B**) Comparing with the model involving only bilateral thalami in Fig. 3, conditioning cortical input significantly reduced the efferent connectivity from thalamus to hand tremor in both DBS off and DBS on conditions. (**C**) Comparing with the model involving only bilateral sensorimotor cortices in Fig. 3, conditioning thalamic input significantly reduced the efferent connectivity from cortex to hand tremor in both DBS off and DBS on conditions. (**D**) Directed afferent connectivity from hand tremor to sensorimotor cortex and VIM thalamus quantified using gOPDC. (**E**) Comparing with the model involving only bilateral thalami in Fig. 3, conditioning cortical input significantly reduced the afferent connectivity from hand tremor to thalamus in both DBS off and DBS on conditions. (**F**) Comparing with the model involving only bilateral sensorimotor cortices in Fig. 3, conditioning thalamic input significantly reduced the afferent connectivity from hand tremor to cortex in both DBS off and DBS on conditions. Here, the connectivity in **A**–**F** was quantified in tremor frequency band. (**G**) Directed connectivity between sensorimotor cortices and the contralateral VIM thalamus relative to the focused hand tremor quantified using gOPDC. (**H**) The directed top-down connectivity from cortex to thalamus (black) was significantly and consistently stronger than bottom-up connectivity from thalamus to cortex (red) in tremor (*left*), alpha (*middle*) and beta (*right*) frequency bands. Bars and error bars indicate mean and standard error of the mean, respectively. Statistics were applied on each comparison using generalized linear mixed-effect modelling on a trial-by-trial basis. Multiple comparisons were corrected by controlling the false discovery rate. ***P < 0.001 after false discovery rate correction. Acc = accelerometer; cCort = contralateral motor cortex; CDBS = continuous deep brain stimulation; cThal = contralateral thalamus; DBS = deep brain stimulation; gOPDC = generalized orthogonalized partial directed coherence; iCort = ipsilateral motor cortex; iThal = ipsilateral thalamus; NC = network-conditioned; VIM = ventral intermediate thalamus.

To investigate the potential relationship between the thalamic-tremor and cortico-tremor networks, we compared the connectivity strength achieved from network-conditioned model (Fig. 4A, NCgOPDC) against those achieved from the gOPDC model involving only thalamic (Fig. 3B) or cortical (Fig. 3E) sources. We found that when conditioning the cortical inputs, the efferent connectivity from thalamus to hand tremor was significantly reduced (Fig. 4B, DBS off, *k* = −0.002 ± 0.001, *P* = 8.75 × 10^−4^; DBS on, *k* = −0.002 ± 0.001, *P* = 9.25 × 10^−6^). Vice versa, conditioning thalamic inputs significantly reduced the efferent connectivity from cortex to hand tremor (Fig. 4C, DBS off, *k* = −0.003 ± 0.001, *P* = 3.57 × 10^−7^; DBS on, *k* = −0.002 ± 0.001, *P* = 2.35 × 10^−6^). Likewise, the afferent connectivity from hand tremor to thalamus (Fig. 4E, DBS off, *k* = −0.004 ± 0.001, *P* = 5.60 × 10^−6^; DBS on, *k* = −0.002 ± 0.001, *P* = 5.05 × 10^−5^) or cortex (Fig. 4F, DBS off, *k* = −0.006 ± 0.001, *P* < 1 × 10^−4^; DBS on, *k* = −0.002 ± 0.001, *P* = 2.67 × 10^−4^) in the network-conditioned model (Fig. 4D) was also significantly reduced compared with the gOPDC model involving only thalamic (Fig. 3B) or cortical (Fig. 3E) sources. These results suggest that the thalamic-tremor and cortico-tremor networks interact with each other, in line with the theory proposed by Raethjen *et al*.^4-6^ When directly comparing the connectivity from thalamus to cortex versus the connectivity from cortex to thalamus (Fig. 4G), we found that the connectivity from cortex to thalamus was significantly stronger than the connectivity in the other direction (from thalamus to cortex; Fig. 4H). The results were similar for tremor (*k* = 0.005 ± 0.001, *P* = 3.60 × 10^−17^), alpha (*k* = 0.007 ± 0.001, *P* = 9.89 × 10^−29^) or beta (*k* = 0.004 ± 4 × 10^−4^, *P* = 9.59 × 10^−23^) frequency bands.

### Connectivity involving contralateral thalamus is positively correlated with DBS effect

**Figure 5:**
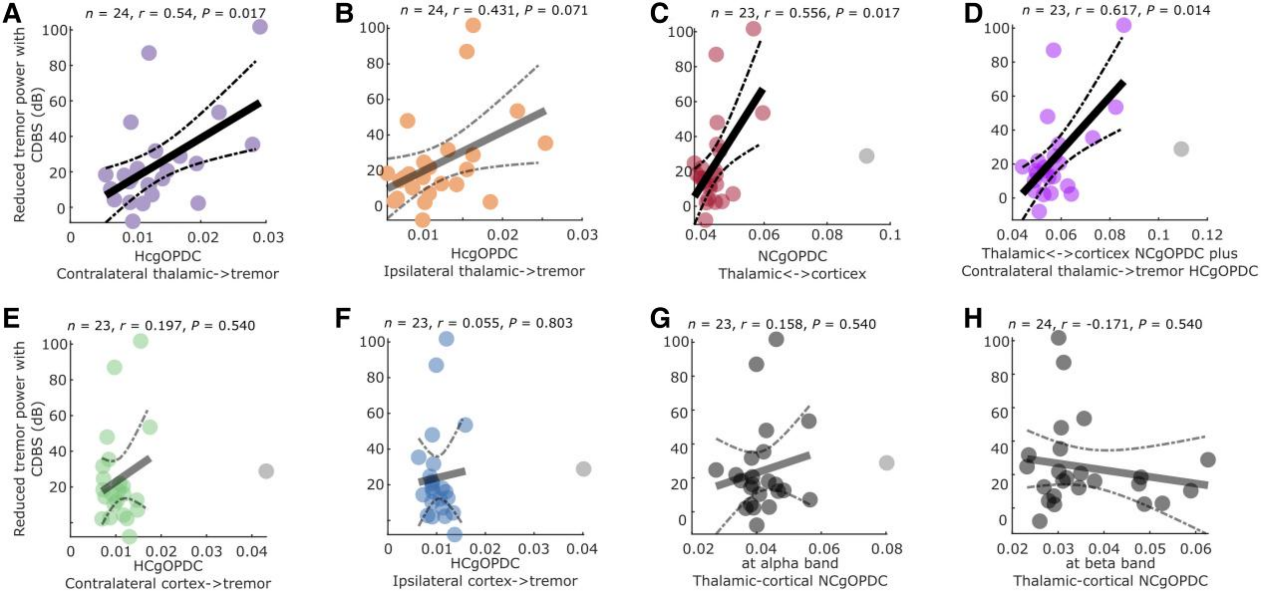
**Correlations between cortico-thalamo-tremor network characteristics and the reduced tremor power with DBS.** (**A** and **B**) Correlations between the efferent connectivity from the contralateral (**A**) or ipsilateral (**B**) thalami with hand tremor and the reduced tremor power with DBS. (**C**) Correlation between the sum of thalamus to cortex and cortex to thalamus connectivity at tremor frequency band and the reduced tremor power with DBS. (**D**) Correlation between the sum of all connectivity at tremor frequency involving the contralateral thalamus and the reduced tremor power with DBS. (**E** and **F**) There was no correlation between the efferent connectivity from the contralateral (**E**) or ipsilateral (**F**) sensorimotor cortices to hand tremor and the reduced tremor power with DBS. (**G** and **H**) There was no correlation between the sum of thalamus to cortex and cortex to thalamus connectivity at alpha (**G**) or beta (**H**) frequency band and the reduced tremor power with DBS. *P*-values were corrected for multiple comparisons by controlling the false discovery rate. CDBS = continuous deep brain stimulation; DBS = deep brain stimulation; gOPDC = generalized orthogonalized partial directed coherence; HC = hemisphere-conditioned.

To investigate further whether the cortico-thalamo-tremor network characteristics could be used to predict the effect on tremor suppression with VIM DBS, we performed Pearson’s correlation analysis between different connectivity measurements and the DBS effect in reducing tremor. This analysis revealed that the efferent connectivity from the contralateral thalamus to hand tremor (Fig. 5A; *r* = 0.54, *P* = 0.017) and the overall connectivity strength between thalamus and cortex at tremor frequency (thalamus to cortex plus cortex to thalamus; Fig. 5C; *r* = 0.556, *P* = 0.017) were positively correlated with the level of tremor power reduction during DBS on. There was a trend of positive correlation between the efferent connectivity from the ipsilateral thalamus and hand tremor, which, however, did not survive multiple comparison correction (Fig. 5B; *r* = 0.431, *P* = 0.071). Combining all connectivity involving the contralateral thalamus increased the effect size of the positive correlation (Fig. 5D; *r* = 0.617, *P* = 0.014). In addition, there was no correlation between the reduced tremor power and the efferent connectivity from either the contralateral (Fig. 5E) or the ipsilateral (Fig. 5F) sensorimotor cortex, or the overall connectivity strength between thalamus and cortex in other frequency bands as control (Fig. 5G, alpha band; Fig. 5H, beta band). When using GLME to predict tremor power using various connectivity measurements (Supplementary Table 3, Model 1), only the connectivity involving the thalamus including efferent connectivity from contralateral (*k* = 94.488 ± 21.8, *P* = 4.571 × 10^−5^) and ipsilateral (*k* = 116.54 ± 24.651, *P* = 1.44 × 10^−5^) thalami to hand tremor, connectivity from the thalamus to cortex (*k* = 88.322 ± 22.94, *P* = 2 × 10^−4^) and connectivity from the cortex to thalamus (*k* = 41.844 ± 16.178, *P* = 0.015) in tremor frequency band showed significant prediction effects, but not the efferent connectivity from the sensorimotor cortex to hand tremor. To test whether the connectivity measurements are simply representations of electrode locations, we quantified the distances between the selected contacts and a sweet spot in VIM for tremor suppression with DBS suggested in a previous study^11^ and correlated them with connectivity measurements and DBS effects. The results showed that the connectivity measurements in Fig. 5A–D were not correlated with the distances between contacts and the tremor sweet spot (Supplementary Fig. 7A–D) but provided better prediction of DBS effects than the distances (Supplementary Fig. 7E).

### Thalamic-tremor connectivity is predicted by tremor characteristics

We then used GLME to test whether the thalamic-tremor connectivity strength can be predicted by tremor characteristics (power and instability). This analysis revealed that stronger tremor power (Supplementary Table 3, Model 2, *k* = 0.0002 ± 3.88 × 10^−5^, *P* = 9.12 × 10^−8^) and smaller tremor amplitude instability (indicating more stable tremor; Supplementary Table 3, Model 2, *k* = −0.007 ± 0.002, *P* = 0.001) together predicted greater connectivity involving the contralateral thalamus. In contrast, stronger tremor power (Supplementary Table 3, Model 3, *k* = −0.001 ± 4 × 10^−4^, *P* < 1 × 10^−4^) and greater connectivity involving the contralateral thalamus (Supplementary Table 3, Model 3, *k* = −0.685 ± 0.236, *P* = 0.004) together predicted smaller tremor amplitude instability, i.e. more stable hand tremor. These results confirmed that there is a clear association between the strength of the functional connectivity involving the contralateral thalamus and tremor characteristics.

### Motor cortex and thalamus have separate pathways in tremor propagation

**Figure 6:**
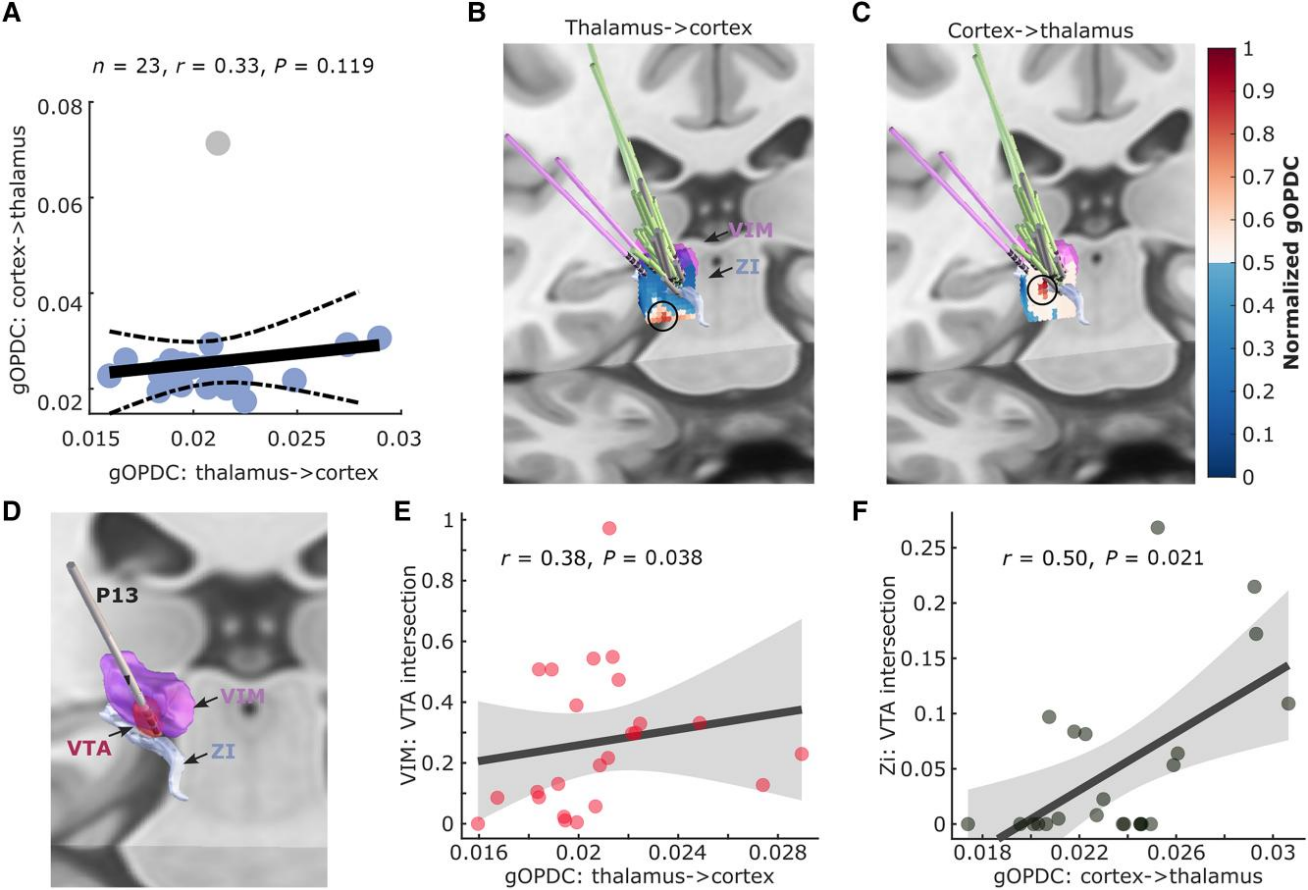
**Comparisons between thalamo-cortical and cortico-thalamic connectivity.** (**A**) Directed connectivity at tremor frequency band (gOPDC) from thalamus to cortex (*x*-axis) was not correlated with that from cortex to thalamus (*y*-axis). (**B** and **C**) The strongest thalamo-cortical (**B**) and cortico-thalamic (**C**) gOPDC clustered at different areas in the standard MNI-152_2009b space. (**D**) A demonstration of the VTA with DBS at 1 mA applied to the selected bipolar local field potential channels (P13). (**E**) Results from Spearman rank correlation between the intersection of the VTA in VIM thalamus and directed connectivity from thalamus to cortex. (**F**) Results from Spearman rank correlation between the intersection of the VTA in ZI and directed connectivity from cortex to thalamus. gOPDC = generalized orthogonalized partial directed coherence; VIM = ventral intermediate thalamus; VTA = volume of tissue activated; ZI = zona incerta.

Although the thalamo-cortical and cortico-thalamic connectivity at tremor frequency predicted the DBS effects (Fig. 5C and D), there was no correlation between them (Fig. 6A). In addition, the strongest thalamo-cortical connectivity and cortico-thalamic connectivity clustered at different areas in the MNI space (Fig. 6B and C). These results suggested that the thalamo-cortical and cortico-thalamic connectivity at tremor frequency band might have different spatial sources. Using Lead-DBS, we quantified the VTA during stimulation at 1 mA for each hemisphere, as shown in Fig. 6D. Correlation analysis revealed that the intersection between VTA and VIM thalamus was positively correlated with the thalamo-cortical connectivity (Fig. 6E; *r* = 0.38, *P* = 0.038), but not with the cortico-thalamic connectivity (*r* = 0.03, *P* = 0.452) measured from the same contacts. In contrast, the intersection between VTA and ZI was positively correlated with the cortico-thalamic connectivity (Fig. 6F; *r* = 0.50, *P* = 0.021), but not with the thalamo-cortical connectivity (*r* = 0.12, *P* = 0.274). The results were consistent when using an amplitude of 2 mA for simulation in Lead-DBS. Together, these results suggest that tremor propagation from the thalamus to motor cortex mainly involves VIM, whereas propagation from the motor cortex back to the thalamus mainly involves ZI/PSA.

## Discussion

**Figure 7:**
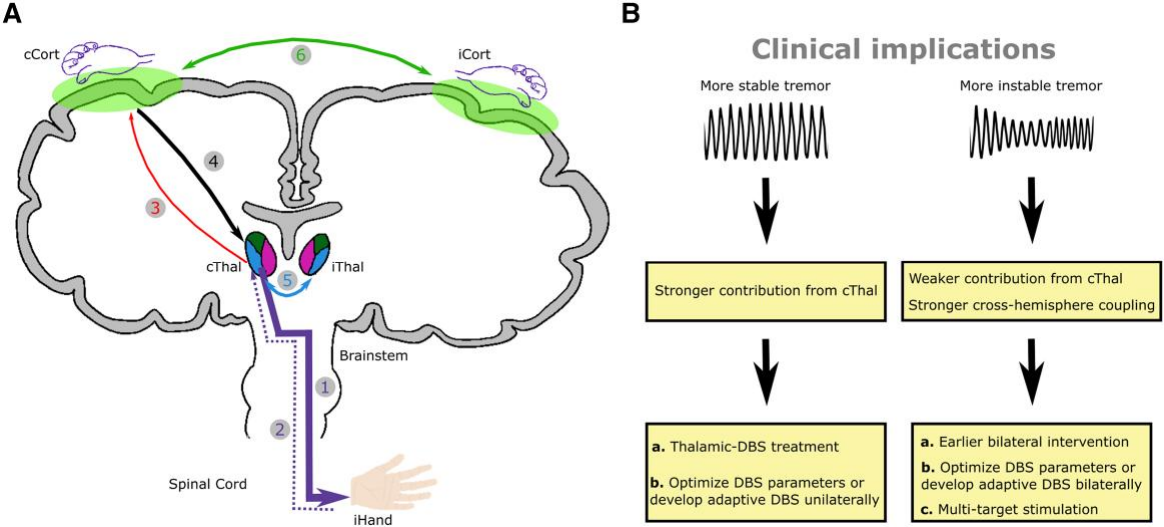
**A summary of the present study.** (**A**) Our study suggests that tremor in essential tremor originates from the contralateral thalamus (path 1). The motor cortex is involved through an indirect pathway, probably via a feedback loop, by receiving afferent input from the tremulous hand through ascending pathways (paths 2 and 3) and sending it back to the thalamus (path 4). There is also significant cross-hemisphere coupling at both subcortical (path 5) and cortical (path 6) levels. (**B**) Potential clinical implications of this study. cCort = contralateral motor cortex; iCort = ipsilateral motor cortex; cThal = contralateral thalamus; DBS = deep brain stimulation; iHand = ipsilateral hand; iThal = ipsilateral thalamus.

In this study, we characterized the cortico-thalamo-tremor network based on hand-acceleration measurements, thalamic LFPs and cortical EEGs recorded simultaneously from people with ET during posture holding in both on and off DBS conditions (Fig. 7). Specifically, we have shown that apart from a stronger lateralized efferent connectivity from the contralateral thalamus to hand tremor (as expected), there is also a significant contribution from the ipsilateral thalamus. The lateral asymmetry was not observed in the cortico-tremor network. Furthermore, although the thalamic-tremor and cortico-tremor networks have different network characteristics and were correlated differently with tremor, they interact with each other. Second, we have shown that both the tremor power during DBS off and the effect of VIM/PSA DBS were predicted only by the connectivity involving the thalamus but not by the cortico-tremor connectivity. In addition, the connectivity involving the contralateral thalamus, which showed the best correlation with the DBS effect, was independently predicted by tremor power and amplitude instability, suggesting that both tremor power and tremor instability represent some level of underlying cortico-thalamo-tremor network characteristics. Lastly, although both thalamo-cortical and cortico-thalamic connectivity at tremor frequency band contributed to predicting DBS effect on tremor suppression, there was no correlation between them, suggesting that the motor cortex and thalamus might have separate pathways in tremor propagation. These results together shed light on the tremor network in ET.

### Verification of the gOPDC connectivity measurements

In this study, the tremor information flow was assessed using partial directed coherence, quantified using a method called gOPDC.^23^ A variant algorithm of this method (without orthogonalization) has also been used to characterize the cerebello-cortical network between essential, Parkinsonian and mimicked tremor.^51^ Results of a few tests provide evidence that the quantified gOPDC measurements are physiologically meaningful: (i) along with the reduction of tremor power during DBS, gOPDC measurements were significantly reduced with DBS compared with during DBS off (Supplementary Table 4), and the laterality of the thalamic-tremor network also disappeared (Supplementary Fig. 5); (ii) we applied gOPDC to surrogate data by shuffling the tremor measurements relative to LFPs and EEGs, and statistical analysis showed that gOPDC measurements based on real data were all significantly bigger than those derived from surrogate data (Supplementary Fig. 8 and the ‘Materials and methods’ section); and (iii) the presented results were still valid when using the variant algorithm without orthogonalization (i.e. gPDC), which resulted in significantly larger connectivity values but had weaker effect sizes in the thalamic laterality and correlation analysis (Supplementary Fig. 9). Please note that the presented thalamic-tremor network laterality phenomenon was not captured by another non-directional connectivity measurement, i.e. imaginary coherence, in which the directionality (i.e. afferent and efferent) and causality are not considered (Supplementary Fig. 10).

### The contralateral thalamus as a main generator of tremor in essential tremor

Existing studies showed that the tremor in ET remains constant when the resonant frequency of the oscillating limb is changed by added inertia.^52,53^ Compared with Parkinsonian tremor, tremor in ET has a much narrower frequency tolerance (a measure that characterizes the temporal evolution of tremor by quantifying the range of frequencies over which the tremor can be considered stable), suggesting that it has a more finely tuned central drive.^13,54,55^ Thalamic neuronal activity was correlated with ET.^56^ Our results showed that only the thalamus-involved connectivity was significantly correlated with both the tremor power during DBS off and the reduced tremor power during DBS on, but not the cortico-tremor connectivity strength. Within the central thalamic-tremor network, the efferent connectivity from the contralateral thalamus to hand tremor was significantly stronger than that from the ipsilateral thalamus. This laterality was not attributable to the selection of analysed bipolar LFP channels, because it persisted when averaging across all bipolar LFP channels within each hemisphere (Supplementary Fig. 11). These results are consistent with existing literature showing strong coherence between thalamic LFP and contralateral muscular EMG in ET^56^ and with clinical evidence demonstrating substantial tremor suppression in the contralateral hand following unilateral thalamic DBS.^57,58^ This evidence suggests that the tremor might originally be generated from the contralateral thalamus. Whaley *et al*.^59^ reported that from a clinical series of 487 consecutive individuals diagnosed with ET, only about half (52%) of the sample reported bilateral initial tremor onset, but eventually ∼90% of the individuals presented bilateral tremor. Here, we also found that there was a significant bidirectional cross-hemisphere coupling within the thalamic-tremor network, highlighted by the significant changes in the efferent and afferent information flow between the contralateral/ipsilateral thalamus and accelerometer when partializing out the contributions from bilateral information flow (Fig. 3C and D). To investigate whether this is physiologically meaningful, we repeated the GLME modelling (Supplementary Table 3) by adding the gOPDC measurements between hemispheres in the models. The results showed that stronger cross-hemisphere communication predicted larger (e.g. power) but more unstable tremor (e.g. larger amplitude and frequency instability) (Supplementary Table 5). In addition, the afferent connectivity from hand tremor back to the ipsilateral thalamus was significantly stronger than that to the contralateral thalamus. However, this was true only for the selected bipolar LFP channels but not when averaging across all bipolar channels within each hemisphere (Supplementary Fig. 11). Together, these results suggest that the ipsilateral thalamus still plays an important role in the development of tremor. Please note that effects of laterality, cross-hemisphere coupling and correlations between thalamic-tremor connectivity and DBS effects were not driven by the fact that most of the patients included in this study presented bilateral dysfunction; our key results were not impacted when partializing out (conditioning) the contribution made by the other tremulous hand (Supplementary Fig. 12).

### Cortical involvement in essential tremor

Conflicting results have been reported on the existence of tremor-related cortical activity in ET.^60,61^ Raethjen *et al*.^6^ reported an intermittent loss of corticomuscular coherence at tremor frequency despite strong peripheral tremor constantly being present. Roy *et al*.^62^ showed that providing high visual feedback worsened tremor compared with low feedback. Here, we found that the strength of the bidirectional cortico-thalamic connectivity predicted baseline tremor power during DBS off (Supplementary Table 3, Model 1), in addition to the effect of DBS (Fig. 5C). Conditioning either cortical or thalamic inputs significantly reduced the thalamic-tremor or cortico-tremor connectivity. These results support the presence of cortical involvement in tremor propagation in ET. In addition, we found that the afferent connectivity from hand tremor back to the cortex was negatively correlated with that to thalamus (Supplementary Table 3, Model 4) and that the connectivity from cortex to thalamus was significantly stronger than the connectivity from thalamus to cortex, with no clear correlation between them (Supplementary Table 3, Model 5; Fig. 6A). Furthermore, we quantified cortico-thalamic and thalamo-cortical gOPDC at the tremor frequency band for each individual bipolar LFP channel for all recorded hemispheres and mapped the values into standard MNI space using the Lead-DBS toolbox. This revealed the strongest cortico-thalamic and thalamo-cortical gOPDC clustered at relatively different areas relative to VIM thalamus (Fig. 6B and C).^11^ Furthermore, simulation analysis revealed that the intersection between the VTA and VIM thalamus was correlated with thalamo-cortical gOPDC, but not cortico-thalamic gOPDC. In comparison, the intersection between the VTA and ZI was correlated with cortico-thalamic gOPDC, but not with thalamo-cortical gOPDC (Fig. 6D–F). There was, however, no correlation between the efferent cortico-tremor connectivity and tremor power or reduced tremor by DBS. Based on these results, we speculate that the cortical involvement in tremor propagation might primarily reflect sensory inputs from the muscles, relayed via ascending tracts such as the dorsal column–medial lemniscus pathway, incorporating the spinal cord and sensory thalamic areas. This process appears relatively independent from the cerebellar outflow pathways, involving the VIM-PSA region, which is likely to be more directly involved in tremor generation and is also a common target for DBS in the treatment of ET.^51,63,64^ Further exploration of this would require new data and is outside the scope of this work.

### Clinical implications

Our results showed that thalamic-tremor connectivity was correlated with the DBS effect on tremor suppression (Fig. 5). Linear mixed-effect modelling revealed that both tremor power and tremor amplitude instability made independent contributions when predicting the directed connectivity involving the contralateral thalamus; more stable tremors were associated with greater connectivity involving the thalamus, which predicted a greater DBS effect. This is consistent with previous studies showing that those with more stable tremors benefitted more from tremor phase-specific DBS targeting the thalamus^65,66^ or phase-specific transcranial electrical stimulation targeting the cerebellum.^14^ Our results also highlighted that more unstable tremor was associated with stronger cross-hemisphere coupling. The outcome of DBS in people with ET is heterogeneous, with some patients not benefitting from the intervention or developing habituation over time. Lead placement might account for some of this heterogeneity in clinical outcomes. However, another important factor to consider is that the clinical syndrome of ET might be underlined by different network characteristics. Indeed, these potential variations in the disease network might necessitate the use of alternative targeting and stimulation modalities. The following clinical implications arise from our study (Fig. 7).

#### Where to stimulate

Thalamic DBS might be more effective for individuals with larger, more stable tremors, because tremors with these characteristics are potentially driven by a more prominent tremor-generating source in the contralateral thalamus. However, our results suggest that unstable tremor arises from a less focal source and is more likely to involve multiple generators, including those in the cortex. This might suggest that more unstable tremors might benefit from alternative surgical targets, such as the PSA or stimulation of multiple regions across the cerebello-thalamo-cortical pathway,^11,67,68^ similar to the strategy that is currently being investigated in chronic pain, involving implantation of electrodes encompassing multiple targets to disrupt the pain network rather than perturbing a single node.^69,70^

#### How to stimulate

Our results show that patients with unstable tremors exhibit stronger cross-hemisphere coupling. This suggests that implanting DBS bilaterally might be more beneficial in these patients, even in the case that tremor might initially present in only one hand. Moreover, when assessing the effects of DBS on a tremulous hand, optimizing stimulation parameters on both sides might be more beneficial than focusing solely on the contralateral side.

#### When to stimulate

Taking into account the variations in the disease network might also be beneficial for the development of a fully embedded closed-loop stimulation system. For instance, for those with more stable tremors, it might be more practical to implement closed-loop stimulation based on the thalamic LFPs,^24^ whereas for those with more unstable tremors, additional sites might be needed for closed-loop stimulation.^71^

### Limitations

There are several limitations in the present study. First, all recordings were conducted 1–6 days after the first surgery for DBS electrode implantations, hence some participants might still experience an appreciable postoperative stun effect, which, however, is more likely to overall reduce rather than increase the effect size of the reported results. Second, although the associations between tremor and tremor network characteristics were explored on a trial-by-trial basis, the correlations between these characteristics and the effect of DBS were investigated only on a hemisphere basis, owing to the lack of data to quantify the reduced tremor in a trial-by-trial basis effectively. Third, although we somehow characterized both thalamic-tremor and cortico-tremor networks, only a thalamus-targeted intervention was applied in this study, hence it is still unclear whether the cortico-tremor network characteristics could be used to predict the effect of cortex-targeted brain stimulation. Furthermore, although tests against surrogate distributions and comparisons between DBS off and on conditions suggest that the cortico-tremor connectivity, quantified based on scalp EEG, is physiologically meaningful, it should be interpreted carefully, and the use of intracranial cortical recordings, such as electrocorticography, should be preferred wherever possible to improve anatomical precision. Finally, we show that the thalamic-tremor network presented both laterality and cross-hemisphere dependency characteristics, but we cannot investigate the potential of using these characteristics to predict the effect of unilateral DBS further, because bilateral stimulation was applied for most of the patients in this study.

## Supplementary Material

awae387_Supplementary_Data

## Data Availability

The data and codes will be shared on the data-sharing platform of the MRC Brain Network Dynamics Unit: https://data.mrc.ox.ac.uk/mrcbndu/data-sets/search.

## References

[1] Brin MF, Koller W. Epidemiology and genetics of essential tremor. Mov Disord. 1998;13:55–63. 10.1002/mds.870131310 9827596

[2] Louis ED, Ferreira JJ. How common is the most common adult movement disorder? Update on the worldwide prevalence of essential tremor. Mov Disord. 2010;25:534–541. 20175185 10.1002/mds.22838

[3] Dallapiazza RF, Lee DJ, De Vloo P, et al Outcomes from stereotactic surgery for essential tremor. J Neurol Neurosurg Psychiatry. 2019;90:474–482. 30337440 10.1136/jnnp-2018-318240 PMC6581115

[4] Raethjen J, Lindemann M, Schmaljohann H, Wenzelburger R, Pfister G, Deuschl G. Multiple oscillators are causing parkinsonian and essential tremor. Mov Disord. 2000;15:84–94. 10634246 10.1002/1531-8257(200001)15:1<84::aid-mds1014>3.0.co;2-k

[5] Raethjen J, Lindemann M, Morsnowski A, et al Is the rhythm of physiological tremor involved in cortico-cortical interactions? Mov Disord. 2004;19:458–465. 15077245 10.1002/mds.10686

[6] Raethjen J, Govindan RB, Kopper F, Muthuraman M, Deuschl G. Cortical involvement in the generation of essential tremor. J Neurophysiol. 2007;97:3219–3228. 17344375 10.1152/jn.00477.2006

[7] Lyons KE, Pahwa R, Busenbark KL, Tröster AI, Wilkinson S, Koller WC. Improvements in daily functioning after deep brain stimulation of the thalamus for intractable tremor. Mov Disord. 1998;13:690–692. 9686776 10.1002/mds.870130414

[8] Obwegeser AA, Uitti RJ, Turk MF, Strongosky AJ, Wharen RE. Thalamic stimulation for the treatment of midline tremors in essential tremor patients. Neurology. 2000;54:2342–2344. 10881269 10.1212/wnl.54.12.2342

[9] Baizabal-Carvallo JF, Kagnoff MN, Jimenez-Shahed J, Fekete R, Jankovic J. The safety and efficacy of thalamic deep brain stimulation in essential tremor: 10 years and beyond. J Neurol Neurosurg Psychiatry. 2014;85:567–572. 24096713 10.1136/jnnp-2013-304943

[10] Cury RG, Fraix V, Castrioto A, et al Thalamic deep brain stimulation for tremor in Parkinson disease, essential tremor, and dystonia. Neurology. 2017;89:1416–1423. 28768840 10.1212/WNL.0000000000004295

[11] Al-Fatly B, Ewert S, Kübler D, Kroneberg D, Horn A, Kühn AA. Connectivity profile of thalamic deep brain stimulation to effectively treat essential tremor. Brain. 2019;142:3086–3098. 31377766 10.1093/brain/awz236

[12] Gironell A, Martínez-Horta S, Aguilar S, et al Transcranial direct current stimulation of the cerebellum in essential tremor: A controlled study. Brain Stimul. 2014;7:491–492. 24582371 10.1016/j.brs.2014.02.001

[13] Brittain JS, Cagnan H, Mehta AR, Saifee TA, Edwards MJ, Brown P. Distinguishing the central drive to tremor in Parkinson’s disease and essential tremor. J Neurosci. 2015;35:795–806. 25589772 10.1523/JNEUROSCI.3768-14.2015 PMC4293424

[14] Schreglmann SR, Wang D, Peach RL, et al Non-invasive suppression of essential tremor via phase-locked disruption of its temporal coherence. Nat Commun. 2021;12:363. 33441542 10.1038/s41467-020-20581-7 PMC7806740

[15] Brittain JS, Probert-Smith P, Aziz TZ, Brown P. Tremor suppression by rhythmic transcranial current stimulation. Curr Biol. 2013;23:436–440. 23416101 10.1016/j.cub.2013.01.068 PMC3629558

[16] Gironell A, Kulisevsky J, Lorenzo J, Barbanoj M, Pascual-Sedano B, Otermin P. Transcranial magnetic stimulation of the cerebellum in essential tremor: A controlled study. Arch Neurol. 2002;59:413–417. 11890845 10.1001/archneur.59.3.413

[17] Popa T, Russo M, Vidailhet M, et al Cerebellar rTMS stimulation may induce prolonged clinical benefits in essential tremor, and subjacent changes in functional connectivity: An open label trial. Brain Stimul. 2013;6:175–179. 22609238 10.1016/j.brs.2012.04.009

[18] Olfati N, Shoeibi A, Abdollahian E, et al Cerebellar repetitive transcranial magnetic stimulation (rTMS) for essential tremor: A double-blind, sham-controlled, crossover, add-on clinical trial. Brain Stimul. 2020;13:190–196. 31624048 10.1016/j.brs.2019.10.003

[19] Hellriegel H, Schulz EM, Siebner HR, Deuschl G, Raethjen JH. Continuous theta-burst stimulation of the primary motor cortex in essential tremor. Clin Neurophysiol. 2012;123:1010–1015. 21982298 10.1016/j.clinph.2011.08.033

[20] Badran BW, Glusman CE, Austelle CW, et al A double-blind, sham-controlled pilot trial of pre-supplementary motor area (Pre-SMA) 1 Hz rTMS to treat essential tremor. Brain Stimul. 2016;9:945–947. 27567469 10.1016/j.brs.2016.08.003 PMC6681894

[21] Reis C, Arruda BS, Pogosyan A, Brown P, Cagnan H. Essential tremor amplitude modulation by median nerve stimulation. Sci Rep. 2021;11:17720. 34489503 10.1038/s41598-021-96660-6 PMC8421420

[22] Shukla AW . Rationale and evidence for peripheral nerve stimulation for treating essential tremor. Tremor Other Hyperkinet Mov (N Y). 2022:12:20. 35949227 10.5334/tohm.685 PMC9205368

[23] Omidvarnia A, Azemi G, Boashash B, O’Toole JM, Colditz PB, Vanhatalo S. Measuring time-varying information flow in scalp EEG signals: Orthogonalized partial directed coherence. IEEE Trans Biomed Eng. 2013;61:680–693. 24144656 10.1109/TBME.2013.2286394

[24] He S, Baig F, Mostofi A, et al Closed-loop deep brain stimulation for essential tremor based on thalamic local field potentials. Mov Disord. 2021;36:863–873. 33547859 10.1002/mds.28513 PMC7610625

[25] Horn A, Li N, Dembek TA, et al Lead-DBS v2: Towards a comprehensive pipeline for deep brain stimulation imaging. Neuroimage. 2019;184:293–316. 30179717 10.1016/j.neuroimage.2018.08.068 PMC6286150

[26] Avants BB, Epstein CL, Grossman M, Gee JC. Symmetric diffeomorphic image registration with cross-correlation: Evaluating automated labeling of elderly and neurodegenerative brain. Med Image Anal. 2008;12:26–41. 17659998 10.1016/j.media.2007.06.004 PMC2276735

[27] Hofmann L, Ebert M, Tass PA, Hauptmann C. Modified pulse shapes for effective neural stimulation. Front Neuroeng. 2011;4:9. 22007167 10.3389/fneng.2011.00009 PMC3181430

[28] Popovych OV, Lysyansky B, Tass PA. Closed-loop deep brain stimulation by pulsatile delayed feedback with increased gap between pulse phases. Sci Rep. 2017;7:1033. 28432303 10.1038/s41598-017-01067-x PMC5430852

[29] Krauss JK, Lipsman N, Aziz T, et al Technology of deep brain stimulation: Current status and future directions. Nat Rev Neurol. 2021;17:75–87. 33244188 10.1038/s41582-020-00426-z PMC7116699

[30] Gilbert Z, Mason X, Sebastian R, et al A review of neurophysiological effects and efficiency of waveform parameters in deep brain stimulation. Clin Neurophysiol. 2023;152:93–111. 37208270 10.1016/j.clinph.2023.04.007

[31] Little S, Pogosyan A, Neal S, et al Adaptive deep brain stimulation in advanced Parkinson disease. Ann Neurol. 2013;74:449–457. 23852650 10.1002/ana.23951 PMC3886292

[32] Debarros J, Gaignon L, He S, et al Artefact-free recording of local field potentials with simultaneous stimulation for closed-loop deep-brain stimulation. In: *2020 42nd Annual International Conference of the IEEE Engineering in Medicine & Biology Society (EMBC)*. IEEE. 2020:3367–3370. 10.1109/EMBC44109.2020.9176665 PMC7116199 33018726

[33] He S, Baig F, Merla A, et al Beta-triggered adaptive deep brain stimulation during reaching movement in Parkinson’s disease. Brain. 2023;146:5015–5030. 37433037 10.1093/brain/awad233 PMC10690014

[34] di Biase L, Brittain JS, Shah SA, et al Tremor stability index: A new tool for differential diagnosis in tremor syndromes. Brain. 2017;140:1977–1986. 28459950 10.1093/brain/awx104 PMC5493195

[35] Allen M, Poggiali D, Whitaker K, Marshall TR, van Langen J, Kievit RA. Raincloud plots: A multi-platform tool for robust data visualization. Wellcome Open Res. 2019;4:4. 31069261 10.12688/wellcomeopenres.15191.1 PMC6480976

[36] Welch P . The use of fast Fourier transform for the estimation of power spectra: A method based on time averaging over short, modified periodograms. IEEE Trans Audio Electroacoust. 1967;15:70–73.

[37] Su D, Zhang F, Liu Z, et al Different effects of essential tremor and Parkinsonian tremor on multiscale dynamics of hand tremor. Clin Neurophysiol. 2021;132:2282–2289. 34148777 10.1016/j.clinph.2021.04.017

[38] Omidvarnia AH, Azemi G, Boashash B, Toole JM, Colditz P, Vanhatalo S. Orthogonalized partial directed coherence for functional connectivity analysis of newborn EEG. In: *Neural Information Processing: 19th International Conference, ICONIP 2012, Doha, Qatar, November 12–15, 2012, Proceedings, Part II 19*. 2012:683–691.

[39] Hipp JF, Hawellek DJ, Corbetta M, Siegel M, Engel AK. Large-scale cortical correlation structure of spontaneous oscillatory activity. Nat Neurosci. 2012;15:884–890. 22561454 10.1038/nn.3101 PMC3861400

[40] Baccala LA, Sameshima K, Takahashi DY. Generalized partial directed coherence. In: *2007 15th International Conference on Digital Signal Processing*. IEEE. 2007:163–166.

[41] Faes L, Nollo G. Extended causal modeling to assess partial directed coherence in multiple time series with significant instantaneous interactions. Biol Cybern. 2010;103:387–400. 20938676 10.1007/s00422-010-0406-6

[42] He S, Deli A, Fischer P, et al Gait-phase modulates alpha and beta oscillations in the pedunculopontine nucleus. J Neurosci. 2021;41:8390–8402. 34413208 10.1523/JNEUROSCI.0770-21.2021 PMC8496192

[43] Maris E, Oostenveld R. Nonparametric statistical testing of EEG- and MEG-data. J Neurosci Methods. 2007;164:177–190. 17517438 10.1016/j.jneumeth.2007.03.024

[44] Lo S, Andrews S. To transform or not to transform: Using generalized linear mixed models to analyse reaction time data. Front Psychol. 2015;6:1171. 26300841 10.3389/fpsyg.2015.01171 PMC4528092

[45] Yu Z, Guindani M, Grieco SF, Chen L, Holmes TC, Xu X. Beyond t test and ANOVA: Applications of mixed-effects models for more rigorous statistical analysis in neuroscience research. Neuron. 2022;110:21–35. 34784504 10.1016/j.neuron.2021.10.030 PMC8763600

[46] Benjamini Y, Hochberg Y. Controlling the false discovery rate: A practical and powerful approach to multiple testing. J R Stat Soc. 1995;57:289–300.

[47] Benjamini Y, Yekutieli D. The control of the false discovery rate in multiple testing under dependency. Ann Stat. 2001:29:1165–1188.

[48] Bain PG, Findley LJ, Atchison P, et al Assessing tremor severity. J Neurol Neurosurg Psychiatry. 1993;56:868–873. 8350102 10.1136/jnnp.56.8.868 PMC1015140

[49] Britton TC, Thompson PD, Day BL, Rothwell JC, Findley LJ, Marsden CD. Rapid wrist movements in patients with essential tremor: The critical role of the second agonist burst. Brain. 1994;117:39–47. 8149213 10.1093/brain/117.1.39

[50] Weerasinghe G, Duchet B, Cagnan H, Brown P, Bick C, Bogacz R. Predicting the effects of deep brain stimulation using a reduced coupled oscillator model. PLoS Comput Biol. 2019;15:e1006575. 31393880 10.1371/journal.pcbi.1006575 PMC6701819

[51] Muthuraman M, Raethjen J, Koirala N, et al Cerebello-cortical network fingerprints differ between essential, Parkinson’s and mimicked tremors. Brain. 2018;141:1770–1781. 29701820 10.1093/brain/awy098

[52] Elble RJ . Physiologic and essential tremor. Neurology. 1986;36:225–225. 3945394 10.1212/wnl.36.2.225

[53] Deuschl G, Krack P, Lauk M, Timmer J. Clinical neurophysiology of tremor. J Clin Neurophysiol. 1996;13:110–121. 8849966 10.1097/00004691-199603000-00002

[54] Brittain JS, Brown P. The many roads to tremor. Exp Neurol. 2013;250:104–107. 24070853 10.1016/j.expneurol.2013.09.012

[55] Hua SE, Lenz FA, Zirh TA, Reich SG, Dougherty PM. Thalamic neuronal activity correlated with essential tremor. J Neurol Neurosurg Psychiatry. 1998;64:273–276. 9489548 10.1136/jnnp.64.2.273 PMC2169953

[56] Pedrosa DJ, Quatuor E-L, Reck C, et al Thalamomuscular coherence in essential tremor: Hen or egg in the emergence of tremor? J Neurosci. 2014;34:14475–14483. 25339758 10.1523/JNEUROSCI.0087-14.2014 PMC6608397

[57] Ondo W, Jankovic J, Schwartz K, Almaguer M, Simpson RK. Unilateral thalamic deep brain stimulation for refractory essential tremor and Parkinson’s disease tremor. Neurology. 1998;51:1063–1069. 9781530 10.1212/wnl.51.4.1063

[58] Huss DS, Dallapiazza RF, Shah BB, Harrison MB, Diamond J, Elias WJ. Functional assessment and quality of life in essential tremor with bilateral or unilateral DBS and focused ultrasound thalamotomy. Mov Disord. 2015;30:1937–1943. 26769606 10.1002/mds.26455

[59] Whaley NR, Putzke JD, Baba Y, Wszolek ZK, Uitti RJ. Essential tremor: Phenotypic expression in a clinical cohort. Parkinsonism Relat Disord. 2007;13:333–339. 17291815 10.1016/j.parkreldis.2006.12.004

[60] Halliday DM, Conway BA, Farmer SF, Shahani U, Russell AJ, Rosenberg JR. Coherence between low-frequency activation of the motor cortex and tremor in patients with essential tremor. Lancet. 2000;355:1149–1153. 10791378 10.1016/s0140-6736(00)02064-x

[61] Hellwig B, Häußler S, Schelter B, et al Tremor-correlated cortical activity in essential tremor. Lancet. 2001;357:519–523. 11229671 10.1016/s0140-6736(00)04044-7

[62] Roy A, Coombes SA, Chung JW, et al Cortical dynamics within and between parietal and motor cortex in essential tremor. Mov Disord. 2019;34:95–104. 30345712 10.1002/mds.27522 PMC8355946

[63] Schuurman PR, Bosch DA, Bossuyt PM, et al A comparison of continuous thalamic stimulation and thalamotomy for suppression of severe tremor. N Engl J Med. 2000;342:461–468. 10675426 10.1056/NEJM200002173420703

[64] Louis ED . Linking essential tremor to the cerebellum: Neuropathological evidence. Cerebellum. 2016;15:235–242. 26129713 10.1007/s12311-015-0692-6

[65] Cagnan H, Pedrosa D, Little S, et al Stimulating at the right time: Phase-specific deep brain stimulation. Brain. 2017;140:132–145. 28007997 10.1093/brain/aww286 PMC5226063

[66] Reis C, He S, Pogosyan A, et al Specific deep brain stimulation revisited: Effects of stimulation on postural and kinetic tremor. *medRxiv*. [Preprint]. doi:10.1101/2022.06.16.22276451

[67] Buijink AW, van der Stouwe AM, Broersma M, et al Motor network disruption in essential tremor: A functional and effective connectivity study. Brain. 2015;138:2934–2947. 26248468 10.1093/brain/awv225

[68] Goede LL, Oxenford S, Kroneberg D, et al Linking invasive and noninvasive brain stimulation in Parkinson’s disease: A randomized trial. Mov Disord. 2024;39:1971–1981. 39051611 10.1002/mds.29940 PMC11568951

[69] Shirvalkar P, Prosky J, Chin G, et al First-in-human prediction of chronic pain state using intracranial neural biomarkers. Nat Neurosci. 2023;26:1090–1099. 37217725 10.1038/s41593-023-01338-z PMC10330878

[70] Shirvalkar P, Starr PA, Chang EF. Ambulatory brain biomarkers of chronic pain: Towards closed loop brain stimulation. Biol Psychiatry. 2024;95:S24.

[71] Opri E, Cernera S, Molina R, et al Chronic embedded cortico-thalamic closed-loop deep brain stimulation for the treatment of essential tremor. Sci Transl Med. 2020;12:eaay7680. 33268512 10.1126/scitranslmed.aay7680 PMC8182660

