# Supplementary material for: Cortico-thalamic tremor circuits and their associations with deep brain stimulation effects in essential tremor
Source: Brain. 2024 Nov 27;148(6):2093–107. doi: 10.1093/brain/awae387 (PMC12129747; doi:10.1093/brain/awae387)
Supplement: awae387_Supplementary_Data [file awae387_supplementary_data.pdf]

Supplementary figures and table

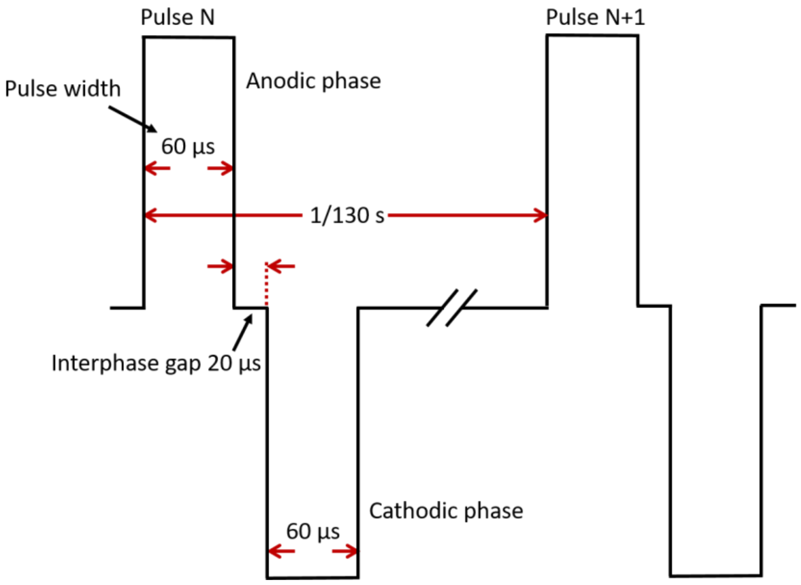

Supplementary Figure 1. A demonstration of some key stimulation parameters used in this study, including a stimulation frequency of 130 Hz, a biphasic pulse width of 60 µs, and an interphase gap of 20 µs.

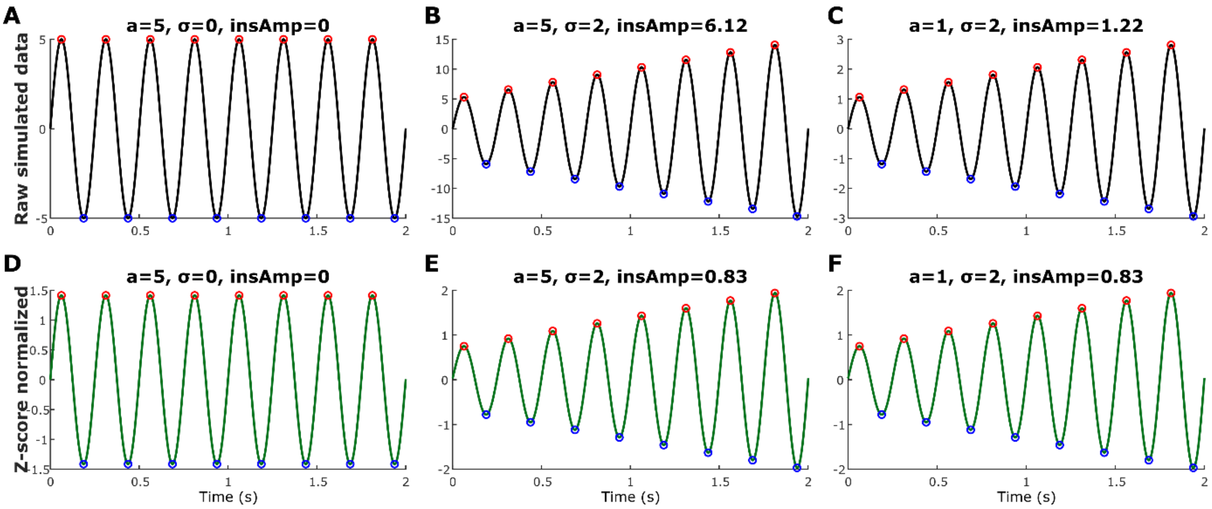

Supplementary Figure 2. Simulation results demonstrate that the tremor instability measurement is not affected by the tremor amplitude following z-score normalization. (A)-(C) Simulated tremor with different amplitudes (a) and changing rates (σ), together with the tremor amplitude instability (insAmp). **(D)-(F)** The same as (A)-(C) but following z-score normalization before quantifying the insAmp. Please note that insAmp was much higher in B with respect to C, even though the changing rate in tremor amplitude was the same. After z-score normalization, insAmp was the same for E and F.

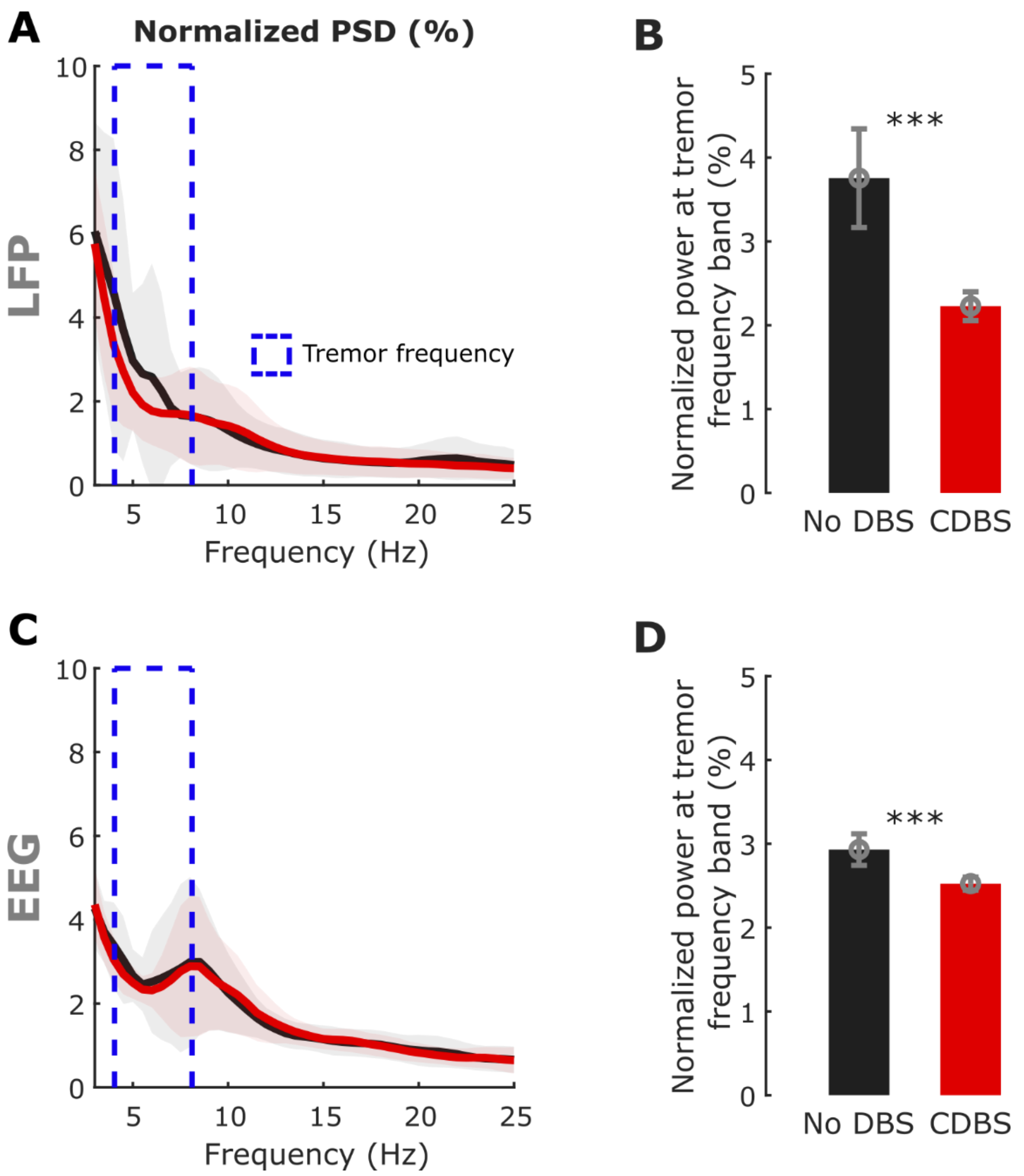

Supplementary Figure 3. Comparisons of power at tremor frequency band in thalamic LFP and cortical EEG between no DBS and CDBS conditions. (A) Normalized power spectral density (PSD) of thalamic LFP in no DBS (black) and CDBS (red) conditions. **(B)** Comparisons of the normalized power in thalamic LFP at tremor frequency band. **(C)** Normalized PSD of cortical EEG in no DBS (black) and CDBS (red) conditions. **(D)** Comparisons of the normalized power in cortical EEG at tremor frequency band. Solid lines in A and C and bars in B and D indicate mean, while shaded areas in A and C and error bars in B and D indicate standard error of the mean (SEM). Statistics were applied between no DBS and CDBS conditions using a nonparametric cluster-based permutation procedure in A and C on a hemisphere basis, or using generalized linear mixed effect modelling in B and D on an individual trial basis. Multiple comparisons were corrected by controlling the false discovery rate (FDR). *** $P_{corrected} < 0.001$.

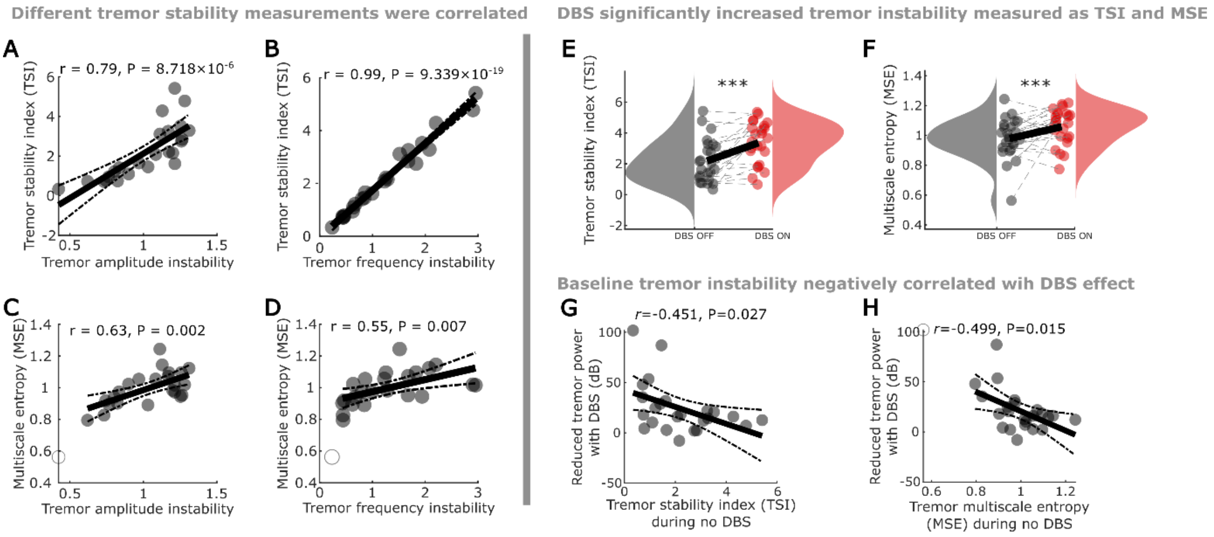

Supplementary Figure 4. (A)-(B) Tremor stability index (TSI) was highly correlated with tremor amplitude (A) and frequency (B) instability used in this study. **(C)-(D)** Tremor multiscale entropy (MSE) was also highly correlated with tremor amplitude (C) and frequency (D) instability. **(E)-(F)** DBS significantly increased tremor instability in terms of TSI (E) and MSE (F). **(G)-(H)** Baseline tremor instability (G: TSI; H: MSE) negatively correlated with the effect of DBS. Correlation analyses were performed using Pearson correlation in plots A-D, G-H. Statistical significance was tested using generalized linear mixed effect modelling on a trial-by-trial basis (E and F). Multiple comparisons were corrected by controlling for the false discovery rate (FDR). *** P < 0.001 after FDR correction.

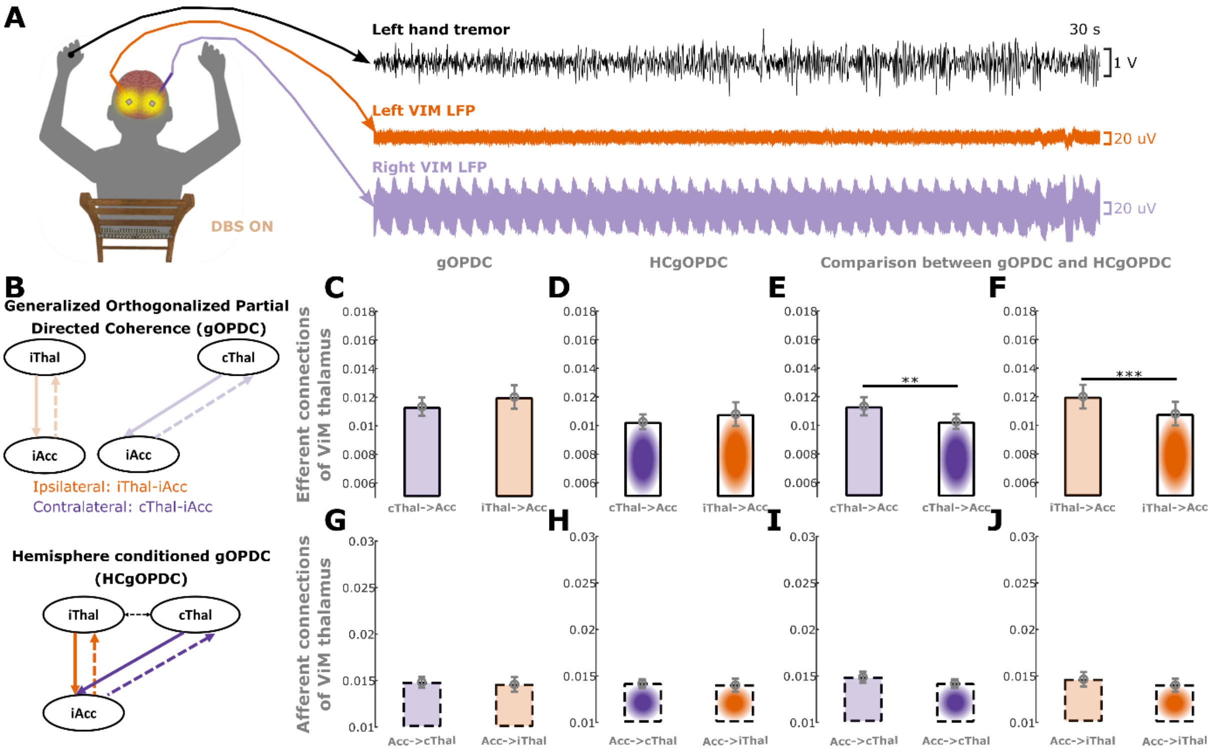

Supplementary Figure 5. Characteristics of thalamic-tremor network during CDBS. (A) A demonstration of left-hand postural tremor and thalamic LFP recordings from patient 1, left hand (P1L) during CDBS condition. **(B)** Directed connectivity between VIM thalamus and limb tremor quantified using generalized Orthogonalized Patial Directed Coherence (gOPDC). Solid lines indicate efferent connectivity from thalamus to limb tremor, while dashed lines indicate afferent connectivity from limb tremor to thalamus. Orange and purple represent the connectivity with ipsilateral and contralateral VIM thalami, respectively. The upper and lower panels indicate gOPDC involving only one thalamus (unconditioned) and both thalami (hemisphere conditioned: HCgOPDC), respectively. **(C)-(D)** There was no significant difference in the efferent connectivity from the contralateral and ipsilateral thalami to limb tremor in either the unconditioned (C) and hemisphere conditioned (D) models. **(E)-(F)** When conditioning the input from the other hemisphere, the efferent connectivity from the contralateral (E) and ipsilateral (F) thalami to limb tremor were both reduced significantly. **(G)-(H)** There was no significant difference in the afferent connectivity from the contralateral and ipsilateral thalami to limb tremor in either the unconditioned (G) and hemisphere conditioned (H) models. **(I)-(J)** When conditioning the input from the other hemisphere, the afferent connectivity from limb tremor to the contralateral (I) and ipsilateral (J) thalami did not change significantly. Bars and error bars indicate mean and standard error of the mean (SEM), respectively. Statistics were applied on each comparison using generalized linear mixed effect

modelling on an individual trial basis. Multiple comparisons were corrected by controlling the false discovery rate (FDR). *** P < 0.001 after FDR correction.

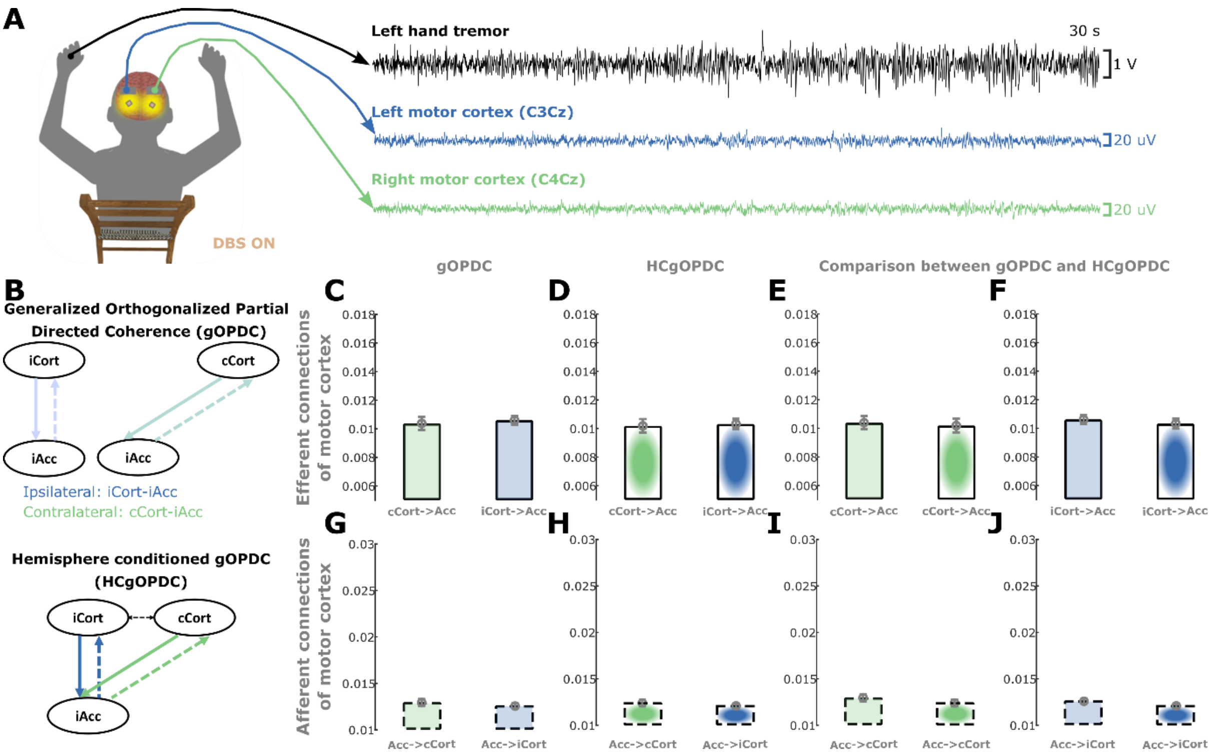

Supplementary Figure 6. Characteristics of cortico-tremor network during CDBS. (A) A demonstration of left-hand postural tremor and cortical EEG recordings from patient 1, left hand (P1L) during no DBS condition. **(B)** Directed connectivity between sensorimotor cortex and limb tremor quantified using generalized Orthogonalized Patial Directed Coherence (gOPDC). Solid lines indicate efferent connectivity from sensorimotor cortex to limb tremor, while dashed lines indicate afferent connectivity from limb tremor to sensorimotor cortex. Blue and green represent the connectivity with ipsilateral and contralateral sensorimotor cortexes, respectively. The upper and lower panels indicate gOPDC involving only one hemisphere (unconditioned) and both hemispheres (hemisphere conditioned: HCgOPDC), respectively. **(C)-(D)** There was no significant difference in the efferent connectivity from the contralateral sensorimotor cortex to limb tremor and the ipsilateral sensorimotor cortex to limb tremor in both unconditioned (C) and hemisphere conditioned (D) models. **(E)-(F)** When conditioning the input from the other hemisphere, the efferent connectivity from the contralateral (E) and ipsilateral (F) sensorimotor cortexes to limb tremor did not change significantly. **(G)-(H)** There was no significant difference in the afferent connectivity from limb tremor to the contralateral and ipsilateral sensorimotor cortexes in both unconditioned (G) and hemisphere conditioned (H) models. **(I)-(J)** When conditioning the input from the other hemisphere, the afferent

connectivity from limb tremor to the contralateral (I) and ipsilateral (J) sensorimotor cortexes did not change significantly. Bars and error bars indicate mean and standard error of the mean (SEM), respectively. Statistics were applied on each comparison using generalized linear mixed effect modelling on an individual trial basis. Multiple comparisons were corrected by controlling the false discovery rate (FDR).

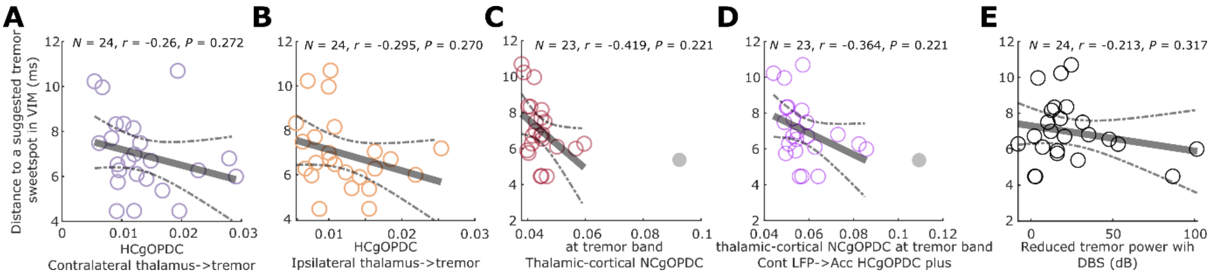

Supplementary Figure 7. Correlation analysis between electrode locations and cortico-thalamo-tremor network characteristics as well as DBS effect. (A)-(D) There was no correlation between the same connectivity measurements as used in Fig. 6A-D and electrode locations measured as distances from contacts to a suggested tremor sweetspot for DBS in VIM (Al-Fatly et al., 2019). **(E)** The distances could not predict DBS effect as good as using connectivity measurements in Fig. 5. Multiple comparisons were corrected by controlling the false discovery rate (FDR).

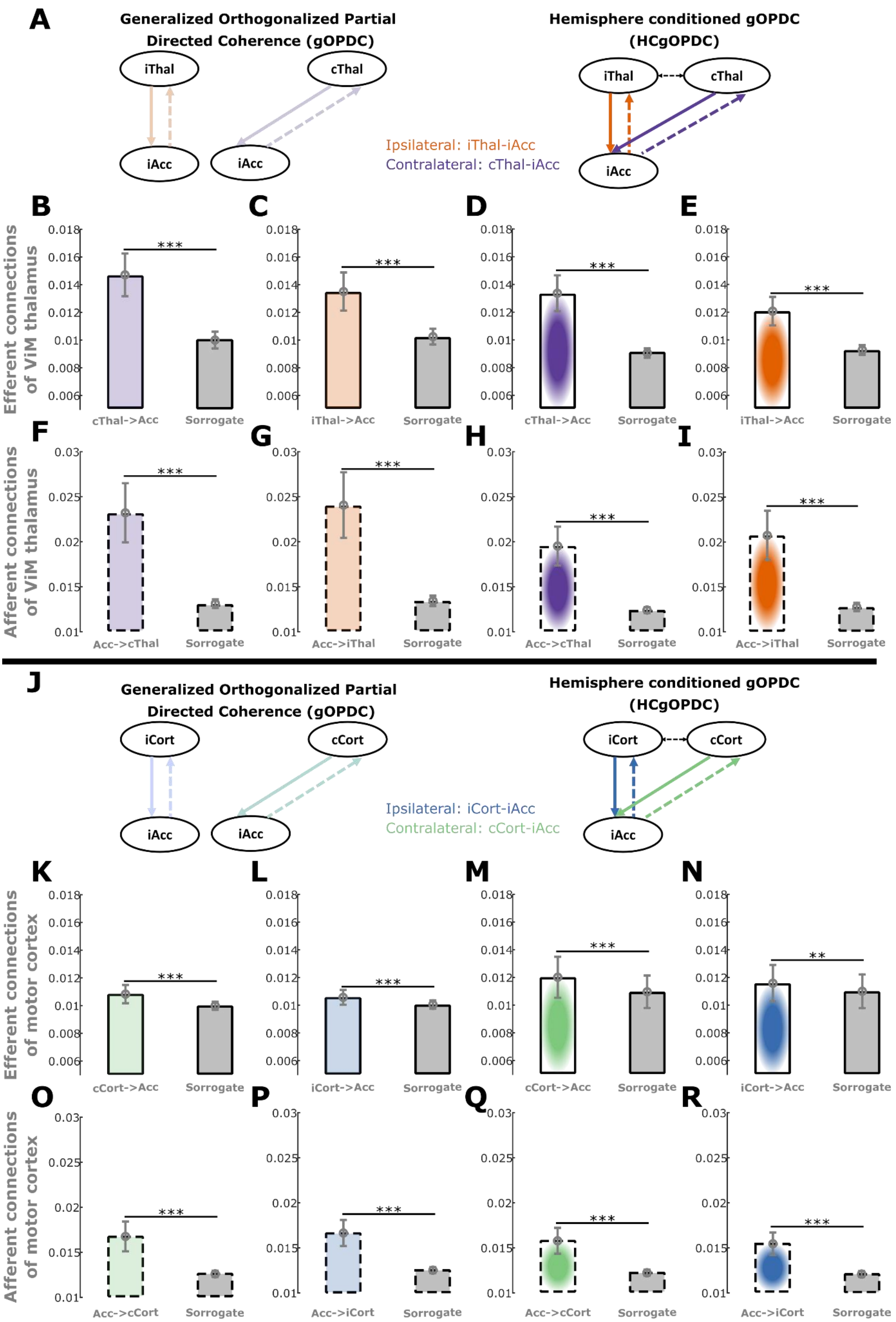

A
Generalized Orthogonalized Partial Directed Coherence (gOPDC)
iThal
cThal
iAcc
iAcc
Hemisphere conditioned gOPDC (HCgOPDC)
Ipsilateral: iThal-iAcc
Contralateral: cThal-iAcc
B
C
D
E
Efferent connection of ViM thalamus
0.018
0.016
0.014
0.012
0.01
0.008
0.006
***
cThal->Acc
iThal->Acc
Sorrogate
F
G
H
I
Afferent connection of ViM thalamus
0.03
0.025
0.02
0.015
0.01
Acc->cThal
Acc->iThal
J
Generalized Orthogonalized Partial Directed Coherence (gOPDC)
iCort
cCort
Hemisphere conditioned gOPDC (HCgOPDC)
Ipsilateral: iCort-iAcc
Contralateral: cCort-iAcc
K
L
M
N
Efferent connection of motor cortex
cCort->Acc
iCort->Acc
**
O
P
Q
R
Afferent connection of motor cortex
Acc->cCort
Acc->iCort

Supplementary Figure 8. Comparisons between the quantified gOPDC measurements (colored) against null distributions (grey). (A) Directed connectivity between VIM thalamus and limb tremor quantified using generalized Orthogonalized Patial Directed Coherence (gOPDC). Solid lines indicate efferent connectivity from thalamus to limb tremor, while dashed lines indicate afferent connectivity from limb tremor to thalamus. Orange and purple represent the connectivity with ipsilateral and contralateral VIM thalami, respectively. The left and right panels indicate gOPDC involving only one thalamus (unconditioned) and both thalami (hemisphere conditioned: HCgOPDC), respectively. **(B)-(E)** Comparisons between real and null efferent gOPDC measurements in the unconditioned (B-C) and hemisphere conditioned (D-E) models. **(F)-(I)** Comparisons between real and null afferent gOPDC measurements in the unconditioned (F-G) and hemisphere conditioned (H-I) models. **(J)-(R)** The same as before but for the gOPDC measurements between cortex and tremor. Bars and error bars indicate mean and standard error of the mean (SEM), respectively. Statistics were applied on each comparison using generalized linear mixed effect modelling on an individual trial basis. Multiple comparisons were corrected by controlling the false discovery rate (FDR). *** $P < 0.001$ after FDR correction.

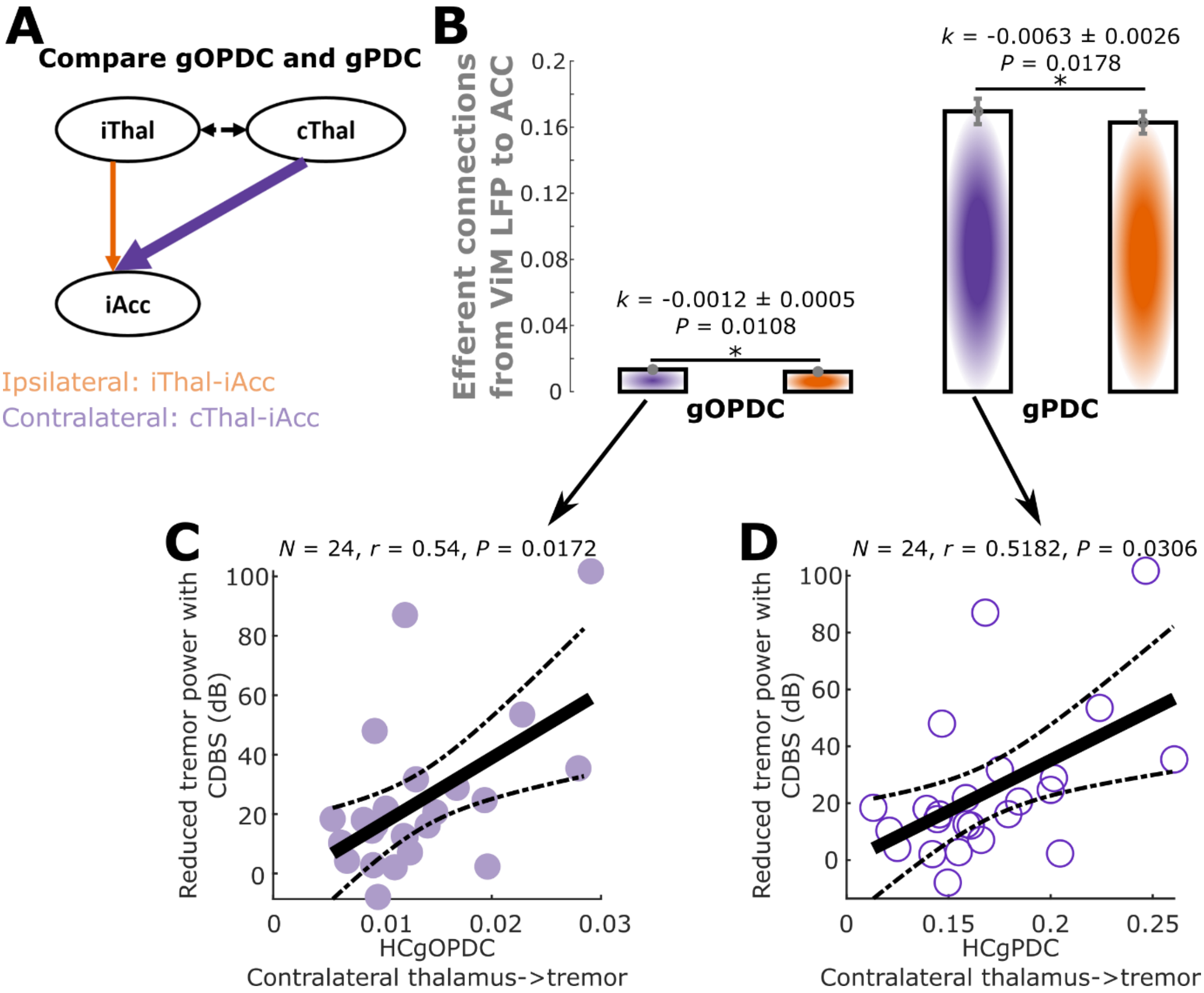

Supplementary Figure 9. Comparison between the utilized gOPDC method against one of its variant methods without orthogonalization (gPDC). (A) Directed connectivity between VIM thalamus and limb tremor quantified using generalized Orthogonalized Patial Directed Coherence (gOPDC) or generalized Patial Directed Coherence (gPDC). Orange and purple represent the connectivity with ipsilateral and contralateral VIM thalami, respectively. Wither purple line indicate stronger connectivity between the contralateral VIM thalamus and tremor as shown in this study. **(B)** The laterality was consistently observed in both gOPDC (left) and gPDC (right) methods, although gOPDC measurements were overall hugely reduced compared with gPDC. **(C-D)** Correlations between the efferent connectivity from the contralateral VIM thalamus to limb tremor (x-axis), measured using gOPDC (C) and gPDC (D), and the reduced tremor power with CDBS (y-axis).

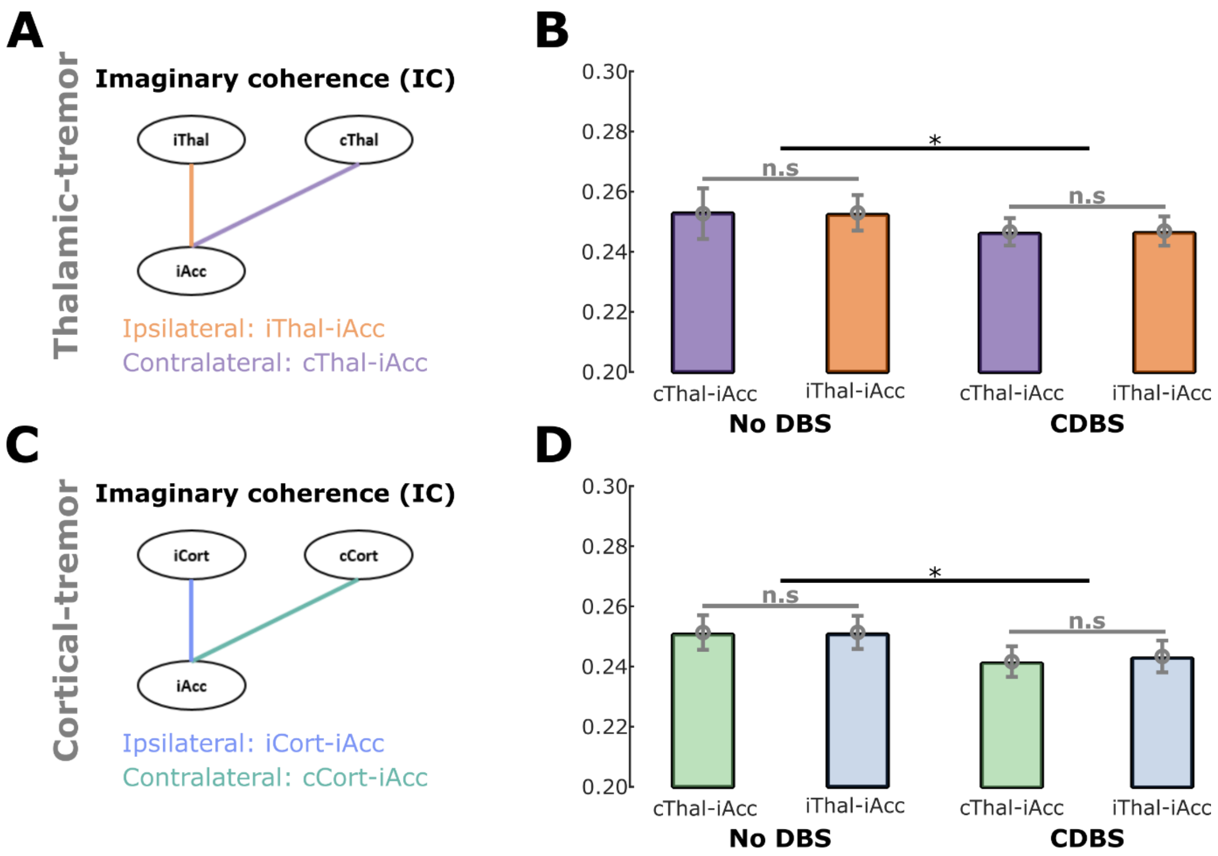

Supplementary Figure 10. Comparisons of imaginary coherence within the thalamic-tremor and cortico-tremor networks. (A) Non-directed connectivity between VIM thalamus and limb tremor quantified using imaginary coherence. Orange and purple represent the connectivity with ipsilateral and contralateral VIM thalami, respectively. **(B)** There was no significant difference in the imaginary coherence between the contralateral thalamic-tremor and ipsilateral thalamic-tremor pairs in either no DBS or CDBS condition, although the imaginary coherence was in general significantly reduced during CDBS compared with no DBS. **(C)** Non-directed connectivity between sensorimotor cortex and limb tremor quantified using imaginary coherence. Blue and green represent the connectivity with ipsilateral and contralateral sensorimotor cortexes, respectively. **(D)** There was no significant difference in the imaginary coherence between the contralateral cortico-tremor and ipsilateral cortico-tremor pairs in either no DBS or CDBS condition, although the imaginary coherence was in general significantly reduced during CDBS compared with no DBS. Bars and error bars indicate mean and standard error of the mean (SEM), respectively. Statistics were applied on each comparison using generalized linear mixed effect modelling on an individual trial basis. Multiple comparisons were corrected by controlling the false discovery rate (FDR). * $P < 0.05$ after FDR correction.

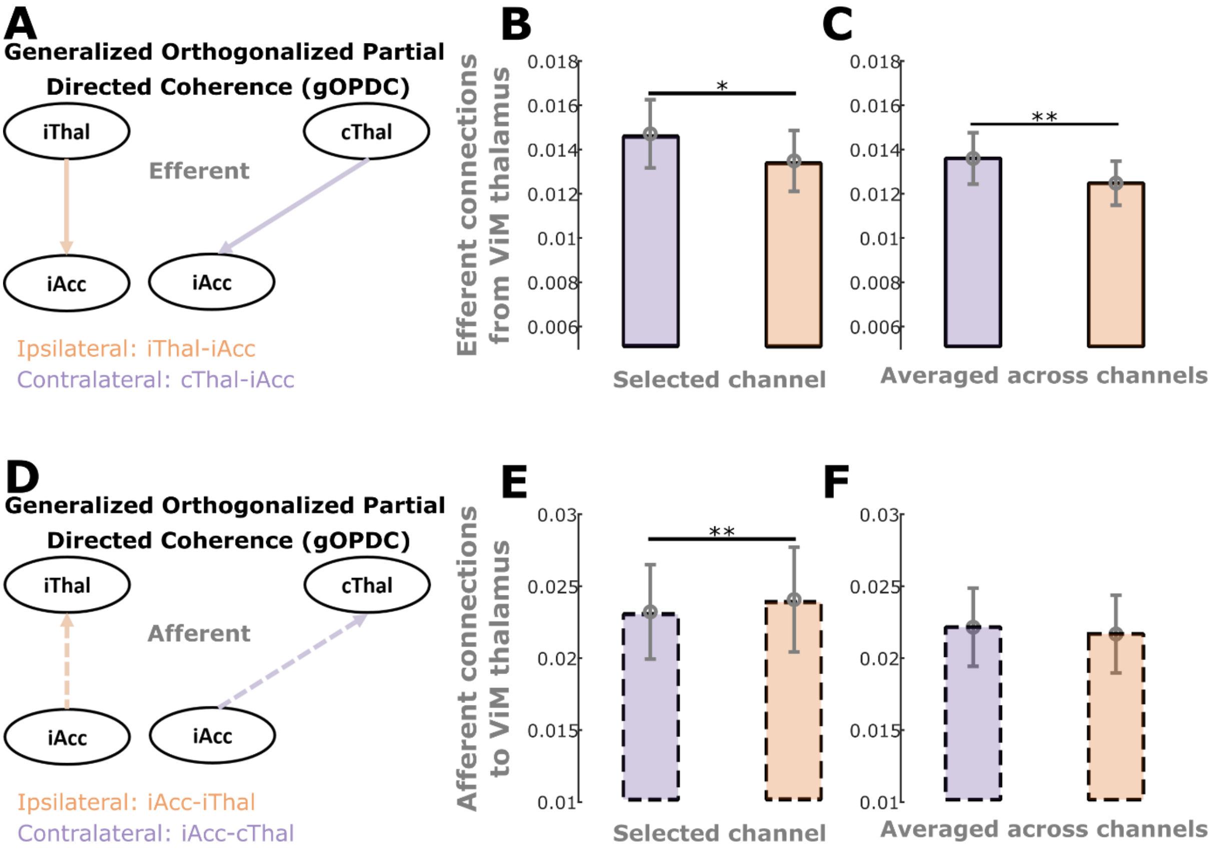

Supplementary Figure 11. Comparisons between gOPDC measurements quantified using the selected bipolar LFP channel and those quantified after averaging across all bipolar LFP channels within each hemisphere. (A) Directed efferent connectivity from VIM thalamus to limb tremor quantified using generalized Orthogonalized Patial Directed Coherence (gOPDC). Orange and purple represent the connectivity from ipsilateral and contralateral VIM thalami, respectively. **(B-C)** Comparisons between gOPDC from the contralateral thalamus to limb tremor and that from the ipsilateral thalamus in the cases of selected LFP channel (B) and averaged across all channels (C). (**D-F**) The same as (A-C) but for the afferent connectivity from limb tremor to VIM thalamus.

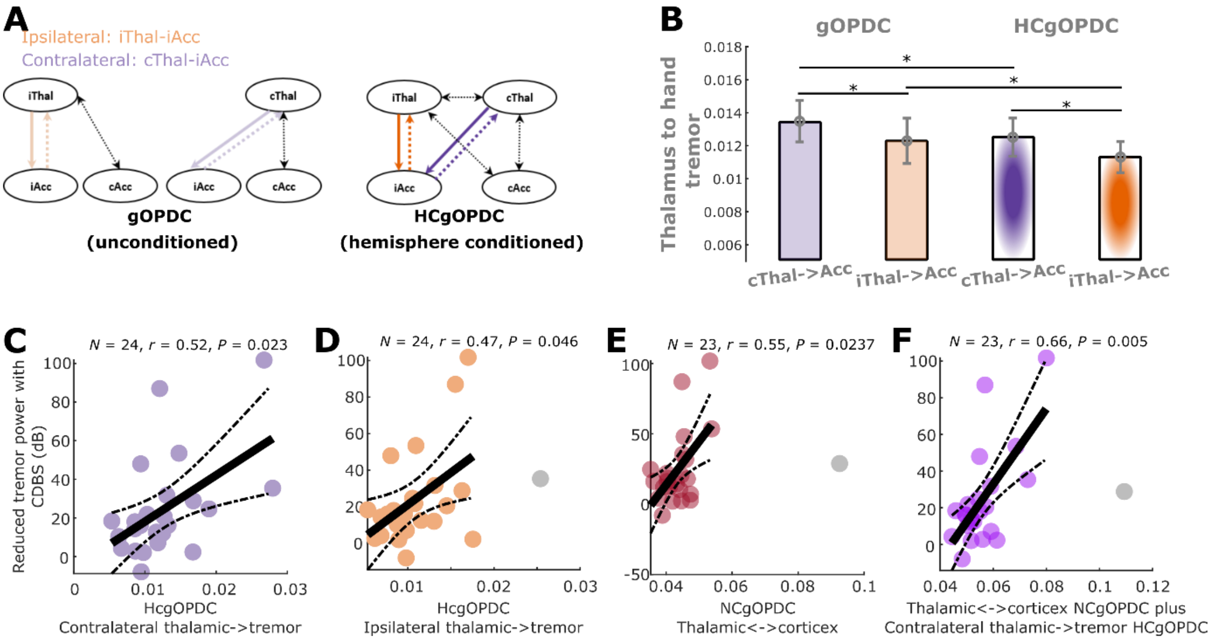

Supplementary Figure 12. The reported results were not caused by the presence of bilateral dysfunction. (A) Directed connectivity between VIM thalamus and hand tremor quantified using generalized Orthogonalized Patial Directed Coherence (gOPDC). Orange and purple represent the connectivity with ipsilateral and contralateral VIM thalami, respectively. The left and right panels indicate gOPDC involving only one thalamus (unconditioned) and both thalami (hemisphere conditioned: HCgOPDC), respectively. Compared with the models in Fig. 3B, here the tremor signal from both hands were included, so that the contribution of the other tremulous hand while comparing the contralateral and ipsilateral thalamic-tremor connectivity for a giving tremulous hand was partialized out. **(B)** Efferent connectivity from the contralateral thalamus was significantly stronger than that from the ipsilateral hemisphere in both unconditioned (left) and hemisphere conditioned (right) models. When conditioning the impact from the other hemisphere, the efferent connectivity from the contralateral (purple) and ipsilateral (orange) thalami to hand tremor were both significantly reduced. **(C)-(D)** Correlations between the efferent connectivity from the contralateral (C) or ipsilateral (D) thalami to hand tremor and the reduced tremor power with DBS. **(E)** Correlation between the sum of thalamus to cortex and cortex to thalamus connectivity at tremor frequency band and the reduced tremor power with DBS. **(F)** Correlation between the sum of connectivity at tremor frequency band involving the contralateral thalamus and the reduced tremor power with DBS. P-values were corrected for multiple comparisons by controlling false discovery rate (FDR). These results were consistent with those reported in Fig. 3 and Fig. 5.

Supplementary Table 1. Effects of laterality, cross-hemisphere coupling, and directionality on thalamic-tremor connectivity

Effects of laterality, cross-hemisphere coupling, and directionality on thalamic-tremor connectivity			
**Model 1**: acclfp* ~ 1 + k_1*latID + k_2*intID + k_3*dirID + k_4*latID*intID + k_5*latID*dirID + k_6*intID*dirID + 1|HemID			
$k_1 = -0.0012 \pm 0.0005$	**$P_1 = 0.020$**	$k_2 = -0.0017 \pm 0.0005$	**$P_2 = 0.002$**
$k_3 = 0.0090 \pm 0.0005$	**$P_3 = 3.955 \times 10^{-61}$**	$k_4 = 0.0001 \pm 0.0006$	$P_4 = 0.868$
$k_5 = 0.0037 \pm 0.0006$	**$P_5 = 2.451 \times 10^{-9}$**	$k_6 = -0.0025 \pm 0.0006$	**$P_6 = 6.540 \times 10^{-5}$**
		AIC = -90969	$R^2 = 0.201$
Effects of laterality on unconditioned (M2) or conditioned (M3) efferent thalamic-tremor gOPDC (Fig. 3C)			
**Model 2**: acclfp^{1} ~ 1 + k*latID + 1|HemID		**Model 3**: acclfp^{2} ~ 1 + k*latID + 1|HemID	
$k = -0.0012 \pm 0.0005$	**$P = 0.029$**	$k = -0.0012 \pm 0.0005$	**$P = 0.004$**
AIC = -24121	$R^2 = 0.127$	AIC = -25371	$R^2 = 0.10$
Effects of cross-hemisphere coupling on gOPDC from the contralateral (M4) or ipsilateral (M5) thalamus to hand tremor (Fig. 3C)			
**Model 4**: acclfp^{3} ~ 1 + k*intID + 1|HemID		**Model 5**: acclfp^{4} ~ 1 + k*intID + 1|HemID	
$k = -0.0016 \pm 0.0005$	**$P = 0.004$**	$k = -0.0017 \pm 0.0005$	**$P = 0.002$**
AIC = -24291	$R^2 = 0.129$	AIC = -25224	$R^2 = 0.113$
Effects of laterality on unconditioned (M6) or conditioned (M7) afferent thalamic-tremor gOPDC (Fig. 3D)			
**Model 6**: lfpacc^{1} ~ 1 + k*latID + 1|HemID		**Model 7**: lfpacc^{2} ~ 1 + k*latID + 1|HemID	
$k = 0.0024 \pm 0.0007$	**$P = 0.001$**	$k = 0.0027 \pm 0.0006$	**$P = 4.732 \times 10^{-5}$**
AIC = -21018	$R^2 = 0.285$	AIC = -22406	$R^2 = 0.196$
Effects of cross-hemisphere coupling on gOPDC from hand tremor to the contralateral (M8) or ipsilateral (M9) thalamus (Fig. 3D)			
**Model 8**: lfpacc^{3} ~ 1 + k*intID + 1|HemID		**Model 9**: lfpacc^{4} ~ 1 + k*intID + 1|HemID	
$k = -0.0043 \pm 0.0006$	**$P = 7.883 \times 10^{-11}$**	$k = -0.004 \pm 0.0007$	**$P = 2.912 \times 10^{-8}$**
AIC = -22469	$R^2 = 0.269$	AIC = -21512	$R^2 = 0.324$

acclfp*=connectivity between thalamus to hand tremor; latID=laterality label (0: contralateral; 1: ipsilateral); intID=cross-hemisphere coupling label (0: unconditioned; 1: conditioned); dirID=directionality label (0: efferent; 1: afferent); acclfp^{1}= efferent thalamic-tremor gOPDC in unconditioned model; acclfp^{2}= efferent thalamic-tremor gOPDC in conditioned model; acclfp^{3}= efferent gOPDC from the contralateral thalamus to hand tremor; acclfp^{4}= efferent gOPDC from the ipsilateral thalamus to hand tremor; lfpacc^{1}= afferent thalamic-tremor gOPDC in unconditioned model; lfpacc^{2}= afferent thalamic-tremor gOPDC in conditioned model; lfpacc^{3}= afferent gOPDC from hand tremor to the contralateral thalamus; lfpacc^{4}= afferent gOPDC from hand tremor to the ipsilateral thalamus. P-values were corrected for multiple comparisons by controlling false discovery rate (FDR) according to the number of comparisons presented in the figure.

Supplementary Table 2. Effects of laterality, cross-hemisphere coupling, and directionality on cortico-tremor connectivity

Effects of laterality, cross-hemisphere coupling, and directionality on cortico-tremor connectivity			
**Model 1**: acceeg* ~ 1 + $k_1$latID + $k_2$intID + $k_3$dirID + $k_4$latID*intID + $k_5$latID*dirID + $k_6$intID*dirID + 1|HemID			
k_1 = -0.0003 ± 0.0004	P_1 = 0.366	k_2 = 0.00014 ± 0.0004	**P_2 = 0.0002**
k_3 = 0.0065 ± 0.0004	**$P_3 = 2.122 \times 10^{-67}$**	k_4 = -0.0002 ± 0.0004	P_4 = 0.636
k_5 = 0.0002 ± 0.0004	P_5 = 0.660	k_6 = -0.0026 ± 0.0004	**$P_6 = 3.481 \times 10^{-9}$**
		AIC = -1.048e+05	R^2 = 0.118
Effects of laterality on unconditioned (M2) or conditioned (M3) efferent cortico-tremor gOPDC (Fig. 3G)			
**Model 2**: acceeg^1 ~ 1 + k*latID + 1|HemID		**Model 3**: acceeg^2 ~ 1 + k*latID + 1|HemID	
k = -0.0004 ± 0.0003	**P = 0.267**	k = -0.0005 ± 0.0004	**P = 0.265**
AIC = - 28652	R^2 = 0.045	AIC = - 26870	R^2 = 0.192
Effects of cross-hemisphere coupling on gOPDC from the contralateral (M4) or ipsilateral (M5) motor cortex to hand tremor (Fig. 3G)			
**Model 4**: acceeg^3 ~ 1 + k*intID + 1|HemID		**Model 5**: acceeg^4 ~ 1 + k*intID + 1|HemID	
k = 0.0014 ± 0.0004	**$P = 9.036 \times 10^{-4}$**	k = 0.0012 ± 0.0004	**P = 0.003**
AIC = -27612	R^2 = 0.103	AIC = -27204	R^2 = 0.069
Effects of laterality on unconditioned (M6) or conditioned (M7) afferent cortico-tremor gOPDC (Fig. 3H)			
**Model 6**: eegacc^1 ~ 1 + k*latID + 1|HemID		**Model 7**: eegacc^2 ~ 1 + k*latID + 1|HemID	
k = -0.0001 ± 0.0005	P = 0.803	k = -0.0004 ± 0.0004	P = 0.511
AIC = -25194	R^2 = 0.181	AIC = -25995	R^2 = 0.157
Effects of cross-hemisphere coupling on gOPDC from hand tremor to the contralateral (M8) or ipsilateral (M9) motor cortex (Fig. 3H)			
**Model 8**: eegacc^3 ~ 1 + k*intID + 1|HemID		**Model 9**: eegacc^4 ~ 1 + k*intID + 1|HemID	
k = -0.0011 ± 0.0005	**P = 0.030**	k = -0.0014 ± 0.0004	**P = 0.007**
AIC = -25475	R^2 = 0.197	AIC = -25823	R^2 = 0.168

acceeg*=connectivity between motor cortex to hand tremor; latID=laterality label (0: contralateral; 1: ipsilateral); intID=cross-hemisphere coupling label (0: unconditioned; 1: conditioned); dirID=directionality label (0: efferent; 1: afferent); acceeg^1= efferent cortico-tremor gOPDC in unconditioned model; acceeg^2= efferent cortical -tremor gOPDC in conditioned model; acceeg^3= efferent gOPDC from the contralateral motor cortex to hand tremor; acceeg^4= efferent gOPDC from the ipsilateral motor cortex to hand tremor; eegacc^1= afferent cortico-tremor gOPDC in unconditioned model; eegacc^2= afferent cortico-tremor gOPDC in conditioned model; eegacc^3= afferent gOPDC from hand tremor to the contralateral motor cortex; eegacc^4= afferent gOPDC from hand tremor to the ipsilateral motor cortex. P-values were corrected for multiple comparisons by controlling false discovery rate (FDR) according to the number of comparisons presented in the figure.

Supplementary Table 3. Generalized linear mixed effect (GLME) modelling

Baseline tremor power (powAcc) was predicted by connectivity involving thalamus, but not cortexes.			
**Model 1**: powAcc ~ 1 + $k_1$acclfp$_1$ + $k_2$acclfp$_2$ + $k_3$acceeg$_1$ + $k_4$acceeg$_2$ + $k_5$eeglfp + $k_6$lfpeeg + 1|HemID			
$k_1 = 94.488 \pm 21.8$	**$P_1 = 4.57 \times 10^{-5}$**	$k_2 = 116.54 \pm 24.651$	**$P_2 = 1.44 \times 10^{-5}$**
$k_3 = -4.326 \pm 29.52$	$P_3 = 0.884$	$k_4 = -44.697 \pm 28.231$	$P_4 = 0.136$
$k_5 = 88.322 \pm 22.94$	**$P_5 = 2 \times 10^{-4}$**	$k_6 = 41.844 \pm 16.178$	**$P_6 = 0.015$**
AIC = 20307		$R^2 = 0.6942$	

Connectivity involving contralateral thalamus was predicted by tremor characteristics			
**Model 2**: (acclfp1+eeglfp+lfpeeg) ~ 1 + $k_1$powAcc + $k_2$irreAmp + $k_3$irreFre + 1|HemID			
$k_1 = 0.0002 \pm 3.88 \times 10^{-5}$	**$P_1 = 9.12 \times 10^{-8}$**	$k_2 = -0.007 \pm 0.002$	**$P_2 = 0.001$**
$k_3 = 0.002 \pm 0.001$	$P_3 = 0.072$	AIC = -9222.6	$R^2 = 0.176$

Tremor amplitude instability was predicted by thalamic-tremor connectivity					
**Model 3**: irreAmp ~ 1 + $k_1$powAcc + k_2(acclfp1+eeglfp+lfpeeg) + 1|HemID					
$k_1 = -0.005 \pm 4 \times 10^{-4}$	**$P_1 < 1$** $\times 10^{-4}$	$k_2 = -0.685 \pm 0.236$	**$P_2 = 0.004$**	AIC = 1979	$R^2 = 0.341$

Afferent tremor-thalamus connectivity negatively correlated with afferent tremor-cortex connectivity			
**Model 4**: lfpacc ~ 1 + $k_1$eegacc + 1|HemID			
$k_1 = -0.184 \pm 0.031$	**$P_1 = 1.68 \times 10^{-9}$**	AIC = -10127	$R^2 = 0.350$

Top-down cortico-thalamic connectivity did not correlate with bottom-up thalamo-cortical connectivity			
**Model 5**: eeglfp ~ 1 + $k_1$lfpeeg + 1|HemID			
$k_1 = 0.014 \pm 0.014$	$P_1 = 0.288$	AIC = -12720	$R^2 = 5.57 \times 10^{-5}$

powAcc=tremor power during DBS OFF; acclfp$_1$=efferent connectivity from the contralateral thalamus to hand tremor; acclfp$_2$=efferent connectivity from the ipsilateral thalamus to hand tremor; acceeg$_1$=efferent connectivity from the contralateral sensorimotor cortex to hand tremor; acceeg$_2$=efferent connectivity from the ipsilateral sensorimotor cortex to hand tremor; eeglfp=directed connectivity from the contralateral thalamus to both cortices at tremor frequency band; lfpeeg=directed connectivity from both cortices to the contralateral thalamus at tremor frequency band; HemID=hemisphere index; AIC=Akaike information criterion; lfpacc=afferent connectivity from hand tremor to both thalami; eegacc=afferent connectivity from hand tremor to both cortices; irreAmp=tremor amplitude instability; irreFre=tremor frequency instability. All models only considered trials during DBS OFF condition. Here acclfp$_1$, acclfp$_2$, acceeg$_1$, acceeg$_2$ were quantified using hemisphere conditioned gOPDC models to take into account the information caused by the mutual source across networks, while eeglfp, lfpeeg, lfpacc, and eegacc were quantified using network conditioned gOPDC models to eliminate the mutual source impact. P-values were corrected for multiple comparisons by controlling false discovery rate (FDR).

Supplementary Table 4. Comparisons of gOPDC measurements between no DBS and CDBS conditions.

Comparisons on hemisphere conditioned gOPDC measurements between no DBS and CDBS			
**Model 1**: acclfp$_1$ ~ 1 + $k_1$stimID + 1|HemID		**Model 2**: acclfp$_2$ ~ 1 + $k_1$stimID + 1|HemID	
k_1 = -0.0034 ± 0.0004	**$P_1 = 3.9968 \times 10^{-15}$**	k_1 = -0.0018 ± 0.0004	**$P_1 = 3.6323 \times 10^{-6}$**
**Model 3**: acceeg$_1$ ~ 1 + $k_1$stimID + 1|HemID		**Model 4**: acceeg$_2$ ~ 1 + $k_1$stimID + 1|HemID	
k_1 = -0.0022 ± 0.0004	**$P_1 = 6.6423 \times 10^{-9}$**	k_1 = -0.0017 ± 0.0004	**$P_1 = 1.3941 \times 10^{-5}$**
**Model 5**: lfpacc$_1$ ~ 1 + $k_1$stimID + 1|HemID		**Model 6**: lfpacc$_2$ ~ 1 + $k_1$stimID + 1|HemID	
k_1 = -0.0061 ± 0.0005	**$P_1 < 0.0001$**	k_1 = -0.0086 ± 0.0006	**$P_1 < 0.0001$**
**Model 7**: eegacc$_1$ ~ 1 + $k_1$stimID + 1|HemID		**Model 8**: eegacc$_2$ ~ 1 + $k_1$stimID + 1|HemID	
k_1 = -0.0038 ± 0.0004	**$P_1 < 0.0001$**	k_1 = -0.0038 ± 0.0004	**$P_1 < 0.0001$**
Comparisons on network conditioned gOPDC measurements between no DBS and CDBS			
**Model 1**: acclfp$_1$ ~ 1 + $k_1$stimID + 1|HemID		**Model 2**: acclfp$_2$ ~ 1 + $k_1$stimID + 1|HemID	
k_1 = -0.0031 ± 0.0004	**$P_1 = 4.4409 \times 10^{-15}$**	k_1 = -0.0021 ± 0.0004	**$P_1 = 2.3928 \times 10^{-8}$**
**Model 3**: acceeg$_1$ ~ 1 + $k_1$stimID + 1|HemID		**Model 4**: acceeg$_2$ ~ 1 + $k_1$stimID + 1|HemID	
k_1 = -0.0014 ± 0.0003	**$P_1 = 2.0727 \times 10^{-5}$**	k_1 = -0.0010 ± 0.0003	**$P_1 = 0.0021$**
**Model 5**: lfpacc$_1$ ~ 1 + $k_1$stimID + 1|HemID		**Model 6**: lfpacc$_2$ ~ 1 + $k_1$stimID + 1|HemID	
k_1 = -0.0053 ± 0.0005	**$P_1 < 0.0001$**	k_1 = -0.0078 ± 0.0006	**$P_1 < 0.0001$**
**Model 7**: eegacc$_1$ ~ 1 + $k_1$stimID + 1|HemID		**Model 8**: eegacc$_2$ ~ 1 + $k_1$stimID + 1|HemID	
k_1 = -0.0019 ± 0.0004	**$P_1 = 1.2974 \times 10^{-7}$**	k_1 = -0.0020 ± 0.0004	**$P_1 = 1.4338 \times 10^{-8}$**
**Model 9**: eeglfp ~ 1 + $k_1$stimID + 1|HemID		**Model 10**: lfpeeg ~ 1 + $k_1$stimID + 1|HemID	
k_1 = -0.0005 ± 0.0005	$P_1 = 0.2774$	k_1 = -0.0020 ± 0.0007	**$P_1 = 0.0026$**

gOPDC=generalized Orthogonalized Patial Directed Coherence; acclfp$_1$=efferent connectivity from contralateral thalamus to limb tremor; stimID=stimulation condition index (0: no DBS, 1: CDBS); HemID=hemisphere index; acclfp$_2$=efferent connectivity from the ipsilateral thalamus to limb tremor; acceeg$_1$=efferent connectivity from the contralateral sensorimotor cortex to limb tremor; acceeg$_2$=efferent connectivity from the ipsilateral sensorimotor cortex to limb tremor; lfpacc1=afferent connectivity from limb tremor to the contralateral thalamus; lfpacc2=afferent connectivity from limb tremor to the ipsilateral thalamus; eegacc1=afferent connectivity from limb tremor to the contralateral sensorimotor cortex; eegacc2=afferent connectivity from limb tremor to the ipsilateral sensorimotor cortex; eeglfp=directed connectivity from the contralateral thalamus to both cortex at tremor frequency band; lfpeeg=directed connectivity from both cortexes to the contralateral thalamus at tremor frequency band.

Supplementary Table 5. Stronger cross-hemisphere communication predicted bigger but more unstable tremor

Baseline tremor power (powAcc) was predicted by connectivity involving thalamus, but not cortices.			
**Model 1**: powAcc ~ 1 + $k_1$acclfp$_1$ + $k_2$acclfp$_2$ + $k_3$acceeg$_1$ + $k_4$acceeg$_2$ + $k_5$eeglfp + $k_6$lfpeeg + $k_7$lfplfp1 + $k_8$lfplfp2 + 1$\vert$HemID			
$k_1 = 100.86 \pm 21.42$	$\mathbf{P_1 = 8.28 \times 10^{-6}}$	$k_2 = 112.9 \pm 24.15$	$\mathbf{P_2 = 8.28 \times 10^{-6}}$
$k_3 = -9.31 \pm 28.92$	$P_3 = 0.748$	$k_4 = -44.96 \pm 27.65$	$P_4 = 0.122$
$k_5 = 97.02 \pm 22.48$	$\mathbf{P_5 = 2.77 \times 10^{-5}}$	$k_6 = 41.81 \pm 15.86$	$\mathbf{P_6 = 0.011}$
$k_7 = 59.83 \pm 13.90$	$\mathbf{P_7 = 2.77 \times 10^{-5}}$	$k_8 = 103.96 \pm 12.84$	$\mathbf{P_8 = 7.08 \times 10^{-15}}$
		AIC = 20214	$R^2 = 0.7065$
Connectivity between two hemispheres was predicted by tremor characteristics			
**Model 2**: (lfplfp1+lfplfp2) ~ 1 + $k_1$powAcc + $k_2$insAmp + $k_3$insFre + 1$\vert$HemID			
$k_1 = 0.0005 \pm 5.08 \times 10^{-5}$	$\mathbf{P_1 = 3.92 \times 10^{-25}}$	$k_2 = 0.007 \pm 0.003$	$\mathbf{P_2 = 0.005}$
$k_3 = 0.004 \pm 0.001$	$\mathbf{P_3 = 0.004}$	AIC = -8101.3	$R^2 = 0.288$
Tremor amplitude instability was predicted by thalamic-tremor connectivity and the connectivity between two hemispheres			
**Model 3**: insAmp ~ 1 + $k_1$powAcc + k_2(acclfp1+eeglfp+lfpeeg) + k_3(lfplfp1+lfplfp2) + 1$\vert$HemID			
$k_1 = -0.0058 \pm 4 \times 10^{-4}$	$\mathbf{P_1 = 9.91 \times 10^{-42}}$	$k_2 = -0.629 \pm 0.236$	$\mathbf{P_2 = 0.008}$
$k_3 = 0.785 \pm 0.184$	$\mathbf{P_3 = 3.05 \times 10^{-5}}$	AIC = 1962.9	$R^2 = 0.345$
Tremor frequency instability was predicted by the connectivity between two hemispheres			
**Model 4**: insFre ~ 1 + $k_1$powAcc + k_2(acclfp1+eeglfp+lfpeeg) + k_3(lfplfp1+lfplfp2) + 1$\vert$HemID			
$k_1 = -0.0177 \pm 9 \times 10^{-4}$	$\mathbf{P_1 = 5.09 \times 10^{-82}}$	$k_2 = 0.358 \pm 0.486$	$\mathbf{P_2 = 0.461}$
$k_3 = 1.732 \pm 0.381$	$\mathbf{P_3 = 8.59 \times 10^{-6}}$	AIC = 5309.4	$R^2 = 0.51$

powAcc=tremor power during DBS OFF; acclfp$_1$=efferent connectivity from the contralateral thalamus to hand tremor; acclfp$_2$=efferent connectivity from the ipsilateral thalamus to hand tremor; acceeg$_1$=efferent connectivity from the contralateral sensorimotor cortex to hand tremor; acceeg$_2$=efferent connectivity from the ipsilateral sensorimotor cortex to hand tremor; eeglfp=directed connectivity from the contralateral thalamus to both cortices at tremor frequency band; lfpeeg=directed connectivity from both cortices to the contralateral thalamus at tremor frequency band; lfplfp1=directed connectivity from the contralateral to the ipsilateral thalami at tremor frequency band; lfplfp2=directed connectivity from the ipsilateral to the contralateral thalami at tremor frequency band; HemID=hemisphere index; AIC=Akaike information criterion; insAmp=tremor amplitude instability; insFre=tremor frequency instability. All models only considered trials during DBS OFF condition. Here acclfp$_1$, acclfp$_2$, acceeg$_1$, acceeg$_2$, lfplfp$_1$, lfplfp$_2$ were quantified using hemisphere conditioned gOPDC models to take into account the information caused by the mutual source across networks, while eeglfp, lfpeeg, lfpacc, and eegacc were quantified using network conditioned gOPDC models to eliminate the mutual source impact. P-values were corrected for multiple comparisons by controlling false discovery rate (FDR).

References:

Al-Fatly B, Ewert S, Kübler D, Kroneberg D, Horn A, Kühn AA. Connectivity profile of thalamic deep brain stimulation to effectively treat essential tremor. Brain. 2019 Oct 1;142(10):3086-98. DOI: 10.1093/brain/awz236
